# Co-expression analysis reveals distinct alliances around two carbon fixation pathways in hydrothermal vent symbionts

**DOI:** 10.1038/s41564-024-01704-y

**Published:** 2024-06-05

**Authors:** Jessica H. Mitchell, Adam H. Freedman, Jennifer A. Delaney, Peter R. Girguis

**Affiliations:** Harvard University, Cambridge, MA USA

**Keywords:** Symbiosis, Marine microbiology, Bacterial transcription, Bacterial systems biology, Biogeochemistry

## Abstract

Most autotrophic organisms possess a single carbon fixation pathway. The chemoautotrophic symbionts of the hydrothermal vent tubeworm *Riftia pachyptila*, however, possess two functional pathways: the Calvin–Benson–Bassham (CBB) and the reductive tricarboxylic acid (rTCA) cycles. How these two pathways are coordinated is unknown. Here we measured net carbon fixation rates, transcriptional/metabolic responses and transcriptional co-expression patterns of *Riftia pachyptila* endosymbionts by incubating tubeworms collected from the East Pacific Rise at environmental pressures, temperature and geochemistry. Results showed that rTCA and CBB transcriptional patterns varied in response to different geochemical regimes and that each pathway is allied to specific metabolic processes; the rTCA is allied to hydrogenases and dissimilatory nitrate reduction, whereas the CBB is allied to sulfide oxidation and assimilatory nitrate reduction, suggesting distinctive yet complementary roles in metabolic function. Furthermore, our network analysis implicates the rTCA and a group 1e hydrogenase as key players in the physiological response to limitation of sulfide and oxygen. Net carbon fixation rates were also exemplary, and accordingly, we propose that co-activity of CBB and rTCA may be an adaptation for maintaining high carbon fixation rates, conferring a fitness advantage in dynamic vent environments.

## Abstract (web summary)

Live tubeworm incubations reveal that chemoautotrophic symbionts regulate the Calvin–Benson–Bassham and reductive tricarboxylic acid pathways to suit geochemistry and metabolism.

## Main

Carbon fixation provides the organic carbon found in all biomass. Six different carbon fixation pathways have been characterized^1^ and more have been proposed^2–4^. Nearly all autotrophic species possess just one known carbon fixation pathway, which typically reflects their evolutionary history and the reduction/oxidation state of their environment^5,6^. Some organisms use carbon fixation machinery for other cellular functions, for example, maintaining intracellular redox balance during fermentation^5^, while others possess multiple enzyme isoforms with varying catalytic properties that are thought to expand their ecological niche^6^. More recently, two carbon fixation pathways were found in the chemoautotrophic endosymbionts of hydrothermal vent tubeworms and closely related free-living bacteria: the Calvin–Benson–Bassham (CBB) and the reverse tricarboxylic acid (rTCA) cycles, both expressed and active^7,8^.

However, we have a limited understanding of (1) whether these pathways are constitutively active and (2) how this activity relates to environmental conditions, and (3) have no understanding of how these pathways integrate with other metabolic processes.

**Fig. 1:**
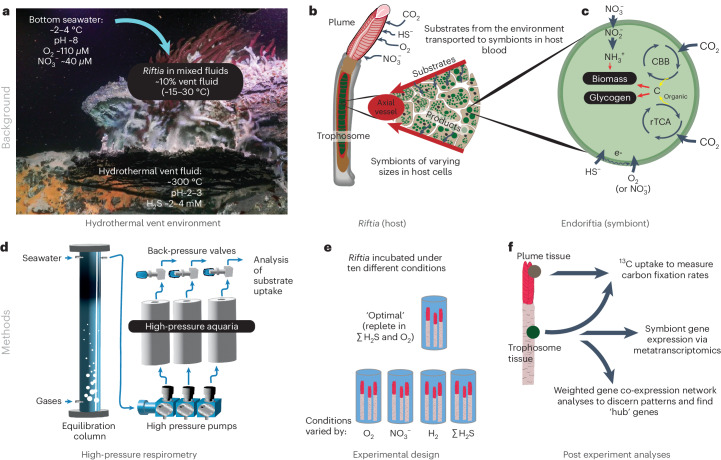
Background, study design and methods used. **a**, The hydrothermal vent habitat of the East Pacific Rise, the ecological niche of the *Riftia* symbiosis. Image courtesy of Schmidt Ocean Institute. **b**, Diagram of the location of Endoriftia within the *Riftia* host. **c**, Simplified illustration of Endoriftia’s substrate utilization. **d**, High-pressure respirometry system, an onboard tool for facilitating experiments under in situ-like conditions. **e**, The conditions that were varied in experiments done on live *Riftia*. **f**, Post-experimental evaluation of *Riftia* tissue and symbiont-containing tissues: ^13^C carbon incorporation, gene expression and co-expression analysis.

Here we studied the vent tubeworm *Riftia pachyptila* (hereafter called *Riftia*) and its endosymbiotic bacteria (‘*Candidatus* Endoriftia persephone’, hereafter called Endoriftia) to investigate how the external environment influences the expression and activity of symbiont CBB and rTCA. Our goals were to (1) discern the relationship between gene/pathway expression and environment, (2) elucidate how these pathways interact with other metabolic processes, and (3) robustly measure the rates of carbon fixation and incorporation. Remarkably, *Riftia* is one of the fastest growing marine invertebrates and can achieve biomass densities comparable to tropical forests^9–11^. *Riftia* resides in diffuse hydrothermal flows where hydrothermal fluid containing hydrogen sulfide (ΣH_2_S: sum of sulfide species H_2_S, HS^−^ and S^2−^), hydrogen (H_2_) and elevated dissolved inorganic carbon (ΣDIC: dissolved species of inorganic carbon) mixes with seawater replete with oxygen (O_2_) and nitrate (NO_3_^−^) (Fig. 1a). This environment is characterized by extreme and rapid shifts in temperature and geochemistry. The symbionts of *Riftia* oxidize ΣH_2_S with O_2_ (and to an extent NO_3_^−^)^12^ to fix carbon, serving as the sole organic carbon source for both symbiont and worm^13^. The symbiont also converts nitrate into bioavailable nitrogen^12^. *Riftia* brings the necessary substrates for these processes from the vent environment to the symbionts via well-developed vasculature, haemoglobin and ion transporters^14^ (Fig. 1b,c).

This organism’s high productivity may be due to the activity of two carbon fixation pathways, but so far there are no data that relate transcript and protein expression to metabolic rate. Also, Endoriftia’s CBB and rTCA pathways are non-canonical. The CBB cycle lacks two essential enzymes that are likely replaced by a pyrophosphate-dependent 6-phosphofructokinase (PPi-PFK)^15^, potentially increasing efficiency by up to 30%^16^. The rTCA genes of Endoriftia are clustered with genes that encode enzymes involved in electron transfers: a NADH dehydrogenase/heterodisulfide reductase (Hdr-Flx) complex potentially involved in flavin-based electron bifurcation (FBEB), an H^+^ translocating NAD(P)^+^ transhydrogenase (encoded by pntAB) and an Na^+^ Rnf membrane complex (hereafter, Rnf)^8^. This clustering suggests energy conservation via FBEB within the rTCA, which could shift the thermodynamic constraints of this pathway. However, while it is tempting to assert that the rTCA pathway has advantages over CBB because it generally requires less ATP per unit carbon fixed, differences in their mechanisms and the oxygen sensitivity of rTCA due to its dependence on reduced ferredoxins (Fd_red_) complicate direct comparisons^17,18^.

Here we present the results from a series of experiments with live *Riftia* in our high-pressure respirometric system. We exposed 30 individuals to a range of environmentally relevant geochemical conditions, with in situ*-*like temperature and pressures. We measured dissolved ΣH_2_S and O_2_ uptake during incubations, directly quantified carbon fixation and incorporation rates using ^13^C-labelled inorganic carbon and sampled individual worms post treatment for metatranscriptomic sequencing to study patterns of differential gene expression (DE). From the latter, we built a co-expression network that provides an in-depth look at an organism that operates two carbon fixation pathways (Fig. 1d–f). We observed patterns of co-expression that reveal both CBB and rTCA as metabolic hubs, with alliances (significant co-expression) to other distinct metabolic processes. Rates of carbon incorporation were also among the highest for autotrophs in natural environments. When seawater ΣH_2_S and/or O_2_ were limited, co-expression analyses implicated the upregulation of the rTCA and a [NiFe] hydrogenase as being among the most significant of metabolic responses. Finally, the distinct bi-modal distribution of other metabolic processes around two functionally degenerate carbon fixation pathways underscores the importance of both and hints at a regulatory independence that may offer more protection from environmental perturbations^19^.

## Results

### *Riftia* substrate uptake rates during experimental treatments

**Fig. 2:**
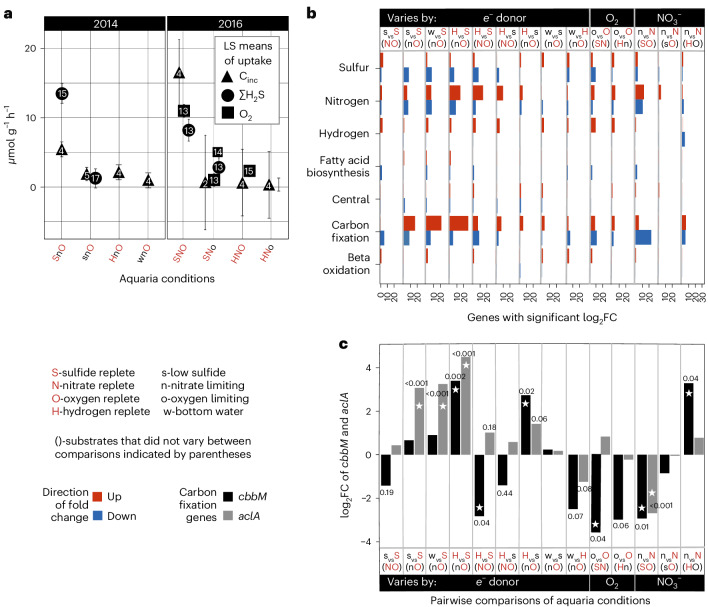
Uptake rates and gene expression under varying conditions. **a**, Symbols represent different measurements: triangles for *C*_inc_ in symbiont-containing tissues, circles for ΣH_2_S and squares for O_2_ uptake rates (O_2_ not measured in 2014). Inside the shapes, numbers indicate the count of independent samples: individual worms for *C*_inc_ and separate sampling events for ΣH_2_S and O_2_. Error bars indicate the standard errors of the least square means. See Suppmentary Table 1 for details on aquaria conditions. **b**, Number of genes that had significant DE for each metabolic category. The log_2_-transformed fold change (log_2_FC) calculated using the limma package in R with a two-sided linear model fit, followed by empirical Bayes moderation of the standard errors (using the eBayes function with the ‘robust=TRUE’ parameter) with a *P*_adj_ ≤ 0.05 (using the Benjamini–Hochberg method for FDR, controlling for multiple comparisons) and a log_2_FC ≥ ∣−1∣. Red bars indicate the number of genes that showed a relative increase in expression, blue bars indicate the number of genes that showed a relative decrease in the two treatments being compared. Comparison of aquaria conditions represented by letters on top row, such that the first letter is the condition that indicates a log fold change (in comparison to the condition represented by the second letter). Letters in parentheses indicate the substrates that did not vary between comparisons. **c**, log_2_FC in *cbbM* and *aclA* (calculated as described above), representative genes for the CBB and the rTCA cycles, respectively. Star indicates *P*_adj_ < 0.05, genes that showed significant DE. Aquaria treatment comparisons are represented by letters at the bottom (see abbreviation key). Source data

The ∑H_2_S and O_2_ uptake rates of *Riftia* during treatments were highly correlated (Fig. 2a and Supplementary Table 2), which is consistent with previous high-pressure respirometric studies^12,20,21^. By contrast, H_2_ uptake rates during treatments were not significantly different from those in control aquaria and did not correlate with O_2_ uptake^22^ (Fig. 2a). Results from statistical tests comparing substrate uptake rates among treatments are reported in Supplementary Table 2.

### *Riftia* carbon incorporation

*Riftia* subjected to experimental treatments showed substantial incorporation of the amended ^13^C-bicarbonate, yielding stable carbon isotope ratios far heavier than the observed natural abundance (*n* = 37; Supplementary Table 1), consistent with an organism that requires both sulfide and oxygen for autotrophic growth. All experimental conditions and rates of ^13^C-labelled inorganic carbon incorporation rates (*C*_inc_) are summarized in Supplementary Table 1. Results of statistical tests comparing *C*_inc_ rates among aquaria are reported in Supplementary Table 2.

### Overall patterns of symbiont gene expression

Of the protein-coding genes, 4.4–15.7% were central carbon genes that are part of either the rTCA or the CBB pathways (many of which are also found in the TCA, pentose phosphate pathways (PPP), glycolysis and gluconeogenesis).

**Extended Data Fig. 1:**
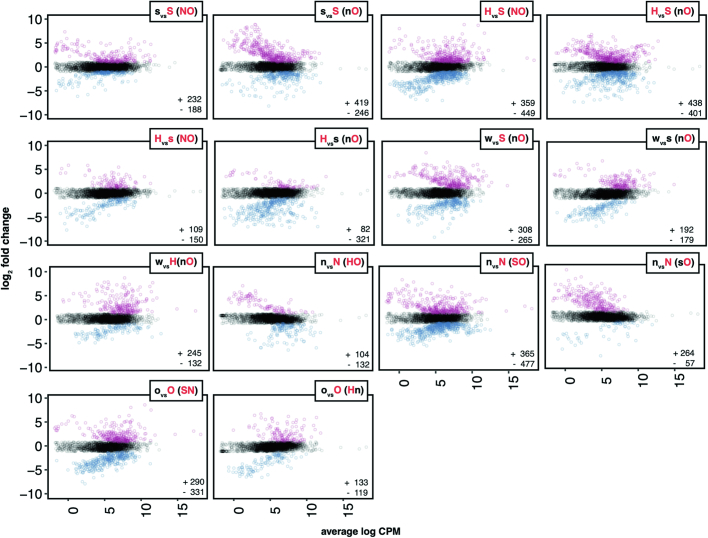
MA plots of each pairwise comparison that showed DE represented by log2 fold change vs. counts per million (CPM). Log_2_ fold change calculated with limma package in R with a two-sided linear model fit followed by empirical Bayes moderation of the standard errors (using the eBayes function with robust = TRUE parameter), controlling for multiple comparisons with a p-adjusted ≤ 0.05 (using the Benjamini-Hochberg method for false discovery rate,). Dots colored in magenta indicate genes that showed a significant relative increase, and dots colored in blue indicate genes that showed a significant relative decrease in expression between aquaria conditions. Black dots indicate genes that did not show significant DE (each plot represent reads that mapped to 3140 genes). Numbers in the corner of each box represent the total number of genes that were DE with +/− indicating log_2_ fold change direction. For details on aquaria conditions, see Fig. 2a and Supplementary Table 1.

Extended Data Fig. 1 shows 14 pairwise comparisons, with significant DE being observed in up to 831 genes. The log-transformed fold change (logFC) versus log-transformed counts per million (CPM) of these comparisons show a wide range of responses, with the highest DE seen in treatments comparing ∑H_2_S to H_2_ (with replete O_2_), as well as in treatments comparing replete NO_3_^−^ to the absence of dissolved NO_3_^−^ (in these cases, ∑H_2_S and O_2_ were both replete).

### DE among functional groups

**Extended Data Fig. 2:**
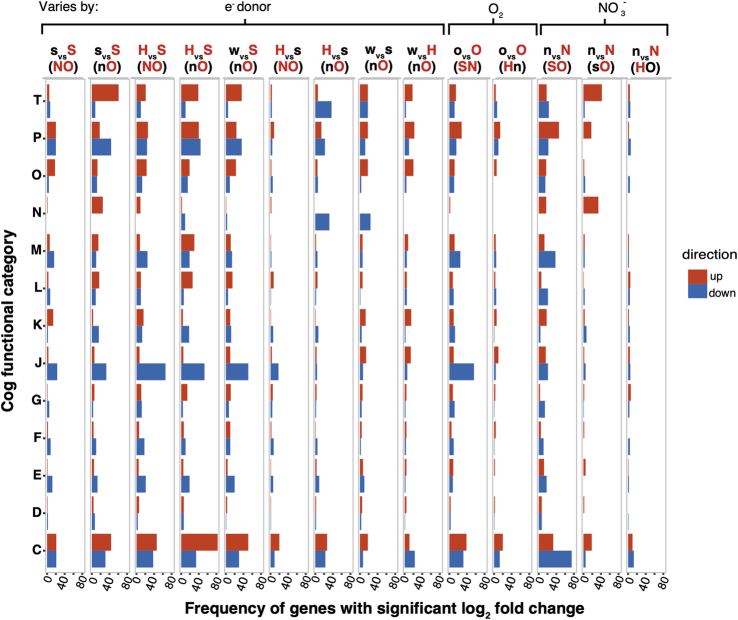
Number of genes that showed significant DE in Cog functional categories. The number of significant DE genes, using a p-adjusted $\le$ 0.05, and a log_2_ fold change ≥ |−1|, grouped according to Cog functional categories. Red bars indicate the number of genes that showed a relative increase in expression, blue bars indicate the number of genes that showed a relative decrease in expression. Log_2_ fold change calculated using limma package in R with a two-sided linear model fit followed by empirical Bayes moderation of the standard errors (using the eBayes function with robust = TRUE parameter) using the Benjamini-Hochberg method for false discovery rate, controlling for multiple comparisons. Comparison of aquaria conditions represented by letters on top row (for abbreviation key see Fig. 1 and Supplementary Table 1). For details on aquaria conditions see Supplementary Table 1. Cog functional category abbreviations listed below: T-Signal transduction L-Replication and repair E-Amino acid metabolism/transport. P-Inorganic ion transport/metabolism K-Transcription D-Cell cycle control and mitosis. O-Post-translational modification/protein turnover J-Translation C-Energy production/conversion. N-Cell motility G-Carbohydrate metabolism/ transport. M-Cell membrane biogenesis. Source data

Genes related to energy (C) and signal transduction (T) exhibited higher DE in treatments with limited or no dissolved ∑H_2_S and/or O_2_, compared with genes involved in translation, ribosome structure and biogenesis (J), as well as inorganic ion transport (P) (Extended Data Fig. 2). The energy (C) category was further sorted: carbon fixation, beta oxidation, nitrogen metabolism, sulfur metabolism, fatty acid biosynthesis and hydrogenases. A comparison of DE among these groups highlighted an increase in DE of carbon fixation genes (some bidirectional, hence could indicate oxidative or anaplerotic reactions), hydrogenases and genes related to nitrogen metabolism in many ∑H_2_S-limiting treatments, accompanied by a relative decrease in expression of sulfur oxidation genes (Fig. 2b).

### DE of key genes involved in the CBB and rTCA cycles

The gene *cbbM* encodes RuBisCO (form II), the carboxylating enzyme of the CBB and ATP citrate lyase (encoded by *aclAB*), which is one of four key enzymes needed to run the TCA cycle in reverse. *aclA* and *cbbM* were selected to represent pathway changes. When dissolved O_2_ was limited, *cbbM* showed a decrease in DE. Conversely, *aclA* exhibited relative increases in DE when dissolved ∑H_2_S was limited or absent (Fig. 2c). Some comparisons showed similar responses for both genes, such as decreased DE when NO_3_^−^ was limiting but ∑H_2_S and O_2_ were replete, and increases in DE of both genes at ∑H_2_S-limiting conditions compared with ∑H_2_S-replete conditions (and in the absence of NO_3_^−^) (Fig. 2c).

### Evidence of energy conservation linked to the rTCA

**Extended Data Fig. 3:**
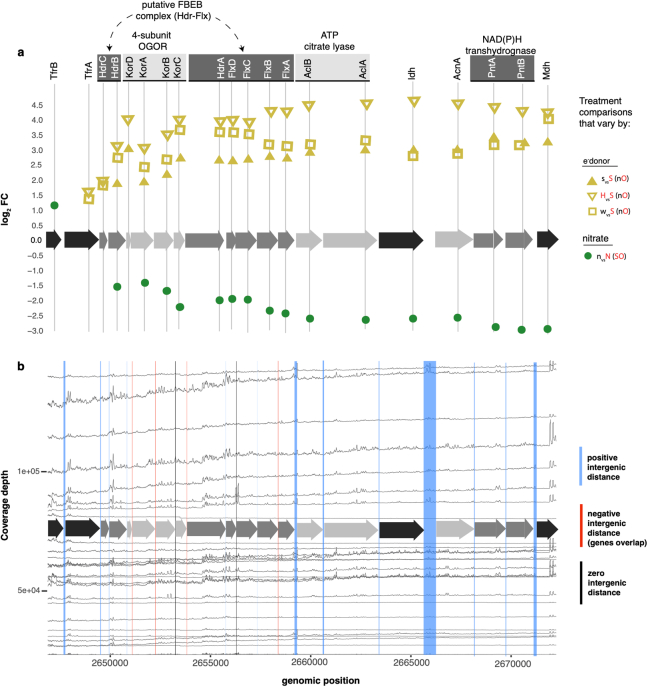
Gene location and expression patterns in the putative rTCA operon. **a**, The log fold change of genes significantly DE on the conserved gene cluster that contains genes of the rTCA, a putative FBEB system (Hdr-Flx), with a four subunit OGOR (KorABCD) in between HdrA and HdrBC), and a transhydrogenase (PntAB) interspersed with other rTCA genes. Arrows on the y = 0 axis indicate genes in the order they appear in the genome. Colors of the shapes indicate the substrate being varied in the comparison, yellow is limiting or no ∑H_2_S compared to ∑H_2_S replete and green is no NO_3_^−^ compared to NO_3_^−^ replete. **b**, Coverage depth in all genomic positions within this gene cluster, including intergenic regions. Positive intergenic regions indicated by blue, whereas red lines indicate that the end of one gene overlaps with the start of another, and black lines indicate that there the intergenic distance is zero.

Genes for ACL and 2-oxoglutarate ferredoxin oxidoreductase (OGOR, encoded by *korABCD*) are essential enzymes of the rTCA cycle. They are found in a gene cluster that also contains a putative flavin-based electron bifurcating (FBEB) complex (Hdr-Flx), which is situated between *aclAB* and *korABCD*, other rTCA/TCA genes and a transhydrogenase (encoded by *pntAB*). This cluster exhibits gene expression patterns that suggest they constitute a functional operon, with similar DE patterns across treatments (except for genes encoding thiol-fumarate reductase (*tfrAB*)). Specifically, these genes ‘increase’ in expression when dissolved H_2_S is limited and ‘decrease’ in expression when dissolved NO_3_^−^ is limiting (Extended Data Fig. 3a). We also see co-expression of the intergenic regions between the genes along this gene cluster, further supporting that it is an operon (Extended Data Fig. 3b). These data provide empirical evidence for an existing theoretical metabolic model^11^, which posits that FBEB using Hdr-Flx may be involved in rTCA carbon fixation where electrons from NADH are shuttled into the rTCA via Hdr-Flx either directly to thiol-fumarate reductase and OGOR or by way of Fd_red_ and a DsrC-like protein.

#### Metabolic alliances revealed via network analysis

**Fig. 3:**
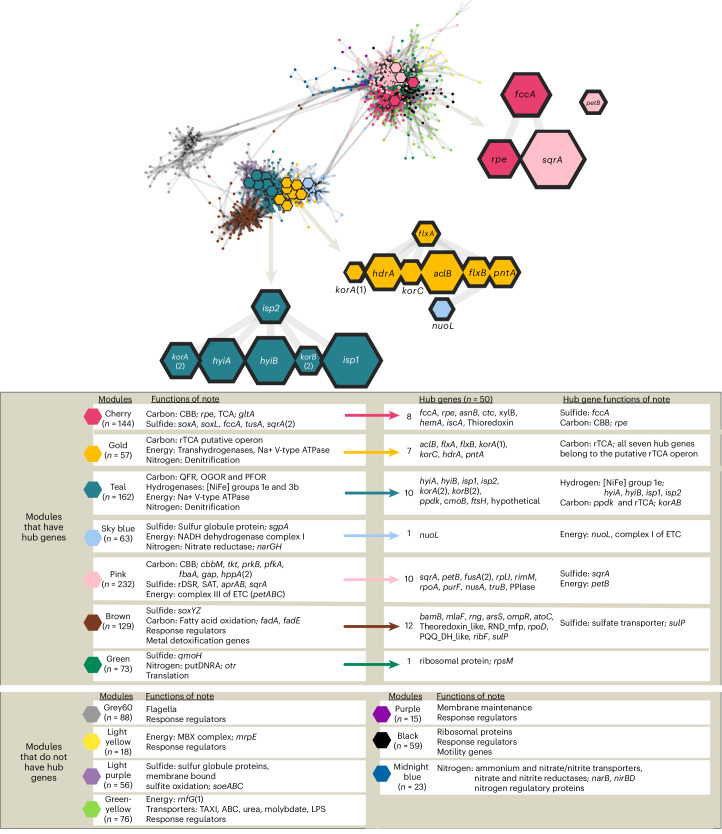
Visualization of network and hub genes made with WGCNA and Cytoscape. The network (top) was made using these filters: edge weight filter >0.05, degree >2 and a nearest neighbour of 10, leaving 1,194 nodes (genes) and 16,271 edges in the network. Each hexagon represents a node, the connections are edges, with hub genes indicated by larger hexagons. Hub genes that were involved in metabolic pathways of interest were zoomed out, proportional to the MCC value. Colours indicate the module grouping for each node. Metabolic functions of note are listed for each module, along with the corresponding hub genes for that module (if present).

**Extended Data Fig. 4:**
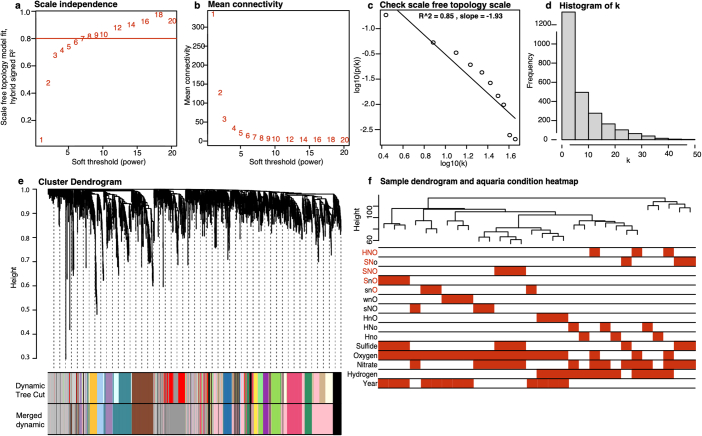
Soft thresholding power determination for WGCNA and module and trait clustering of the WGCNA network. **a**, The scale free fit index for soft thresholding powers. **b**, Mean connectivity as a function of soft thresholding powers. **c**, Scale free topology of frequency distribution of connectivity p(k) when the soft thresholding power is β = 8. **d**, Histogram of mean connectivity when the soft thresholding power is β = 8. **e**, Gene dendrogram with colors along the bottom indicating module color assignment using hierarchical clustering based on TOM dissimilarity (top color row). Bottom color row is module assignment after using a dynamic splicing method, merging highly connected modules with branches reaching below 0.3. **f**, Sample dendrogram and trait heatmap. The leaves of the tree correspond to samples from individual *Riftia*. Red color bands correspond to aquaria conditions in aquaria listed on left (for aquaria conditions and abbreviation key see Fig.1 and Supplementary Table 1). Bottom rows represent conditions merged according to substrate represented by sulfide, hydrogen, oxygen and nitrate. The last row indicates the year these experiments occurred (red represents 2014, white represents 2016).

Module membership and network visualization revealed a clear grouping of genes with associated metabolic systems. Foremost, genes from the rTCA gene cluster, along with hydrogenases, type 2 V-type ATPases and genes associated with denitrification grouped together in the gold and teal modules. The genes for the CBB cycle, reverse dissimilatory sulfate reductase system (rDSR), sulfur oxidizing system (Sox) and periplasmic sulfide oxidation (such as *fccA* and *sqrA*) grouped together in the pink and cherry modules. Most of the genes for motility were clustered in one module (grey60) and shared only few connections with the rest of the network (Fig. 3). The preprocessing and analysis of data structure for this co-expression network can be found in Extended Data Fig. 4a–f.

### DE in network and hub genes

**Extended Data Fig. 5:**
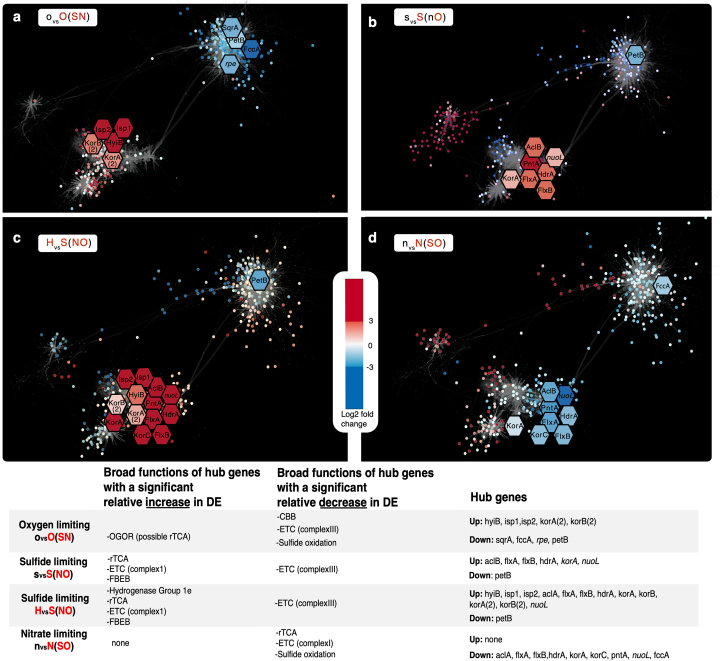
Differential expression patterns overlaid on network in four pairwise comparisons. Differentially expressed (DE) nodes (genes) in co-expression network in four treatment comparisons where the limiting condition was compared to the replete: **a**, Oxygen limiting **b**, Sulfide limiting **c**, Sulfide limiting with hydrogen and **d**, Nitrate limiting. Only nodes that represented genes DE with a p-adjusted < 0.05 are shown, calculated using limma package in R with a two-sided linear model fit followed by empirical Bayes moderation of the standard errors (using the eBayes function with robust = TRUE parameter) and the Benjamini-Hochberg method for false discovery rate, controlling for multiple comparisons. Red indicates a relative increase in expression and blue indicates a relative decrease in expression using a log2 fold scale. Enlarged nodes indicate relevant metabolic genes that were DE and classified as hub genes in cytoHubba.

DE followed network topology (Extended Data Fig. 5a–d); for example, when hub genes had DE, there were concomitant responses in neighbouring genes. The hub genes of the rTCA, FBEB system, PntAB and a group 1e hydrogenase (Hyd1e) all had increased DE under ∑H_2_S and O_2_ limitation (Fig. 2a and Supplementary Table 2). The CBB gene for ribulose-phosphate 3-epimerase (Rpe, encoded by *rpe*) is a hub gene (notably, *cbbM* is not). Rpe catalyses the interconversion of Ru5P and Xu5P, a key step in the regeneration of RuBP. While it can also be involved in the PPP, other genes involved in the CBB (*cbbM*, *tkt*, *gap* and *prkB*) followed the pattern of this hub gene by also showing a decrease in DE at low O_2_ conditions. Under limited dissolved ∑H_2_S, key electron transport chain (ETC) genes—*nuoL* (NADH dehydrogenase, complex I of the ETC) and *petB* (cytochrome *bc*_1_, complex III)—show opposite expression patterns, with *nuoL* increasing and *petB* decreasing. *petB* is in the pink module, along with CBB and sulfide oxidation genes, while nuoL is part of the light blue module, linking to the gold and teal modules associated with the rTCA and Hyd1e pathways. Genes of the rDSR (involved in sulfide oxidation) were relatively decreased under limiting conditions of oxygen and sulfide (similar to their hub neighbours *petB*, *rpe*, *sqrA* and *fccA*). The genes involved in denitrification (*norCB*, *nosZ* and *nirS*) had higher levels of expression under sulfide limitation (similar to their hub neighbours in the rTCA). For a complete list of genes, their DE patterns and network relationships, see GEO series accession no. GSE249345.

### Analysis finds bi-modal distribution of metabolic processes

**Fig. 4:**
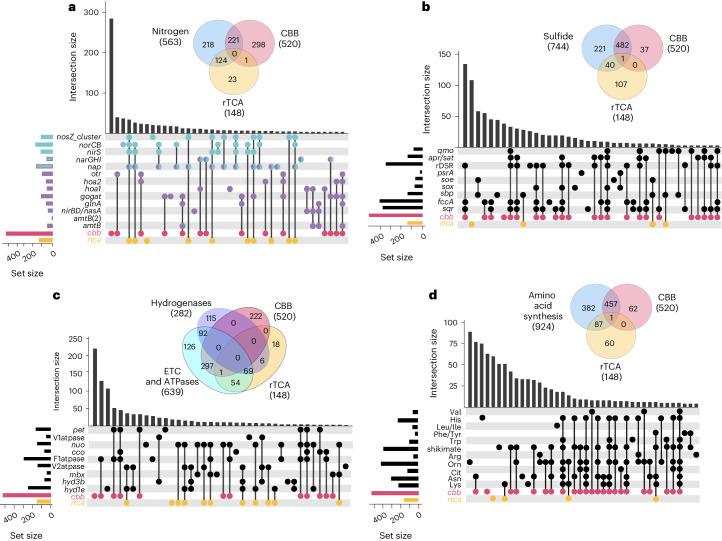
First-neighbour intersections between the CBB, the rTCA and other metabolic functions in the co-expression network. Venn diagrams illustrate the shared first neighbours between broad functional categories and the rTCA and CBB. Beneath each diagram, intersecting set plots detail contributions from pathways and/or functions. Cherry dots signify the CBB, while gold dots denote the rTCA. Columns with overlapping dots highlight intersections of shared neighbours in the network. **a**, Nitrogen metabolism intersections, where purple dots represent assimilatory processes, blue dots symbolize dissimilatory processes, and dual-coloured dots indicate possible utilization in both functions. **b**, Sulfide metabolism intersections. **c**, Hydrogen metabolism, ETC and ATPases. **d**, Amino acid metabolism intersections. Source data

**Fig. 5:**
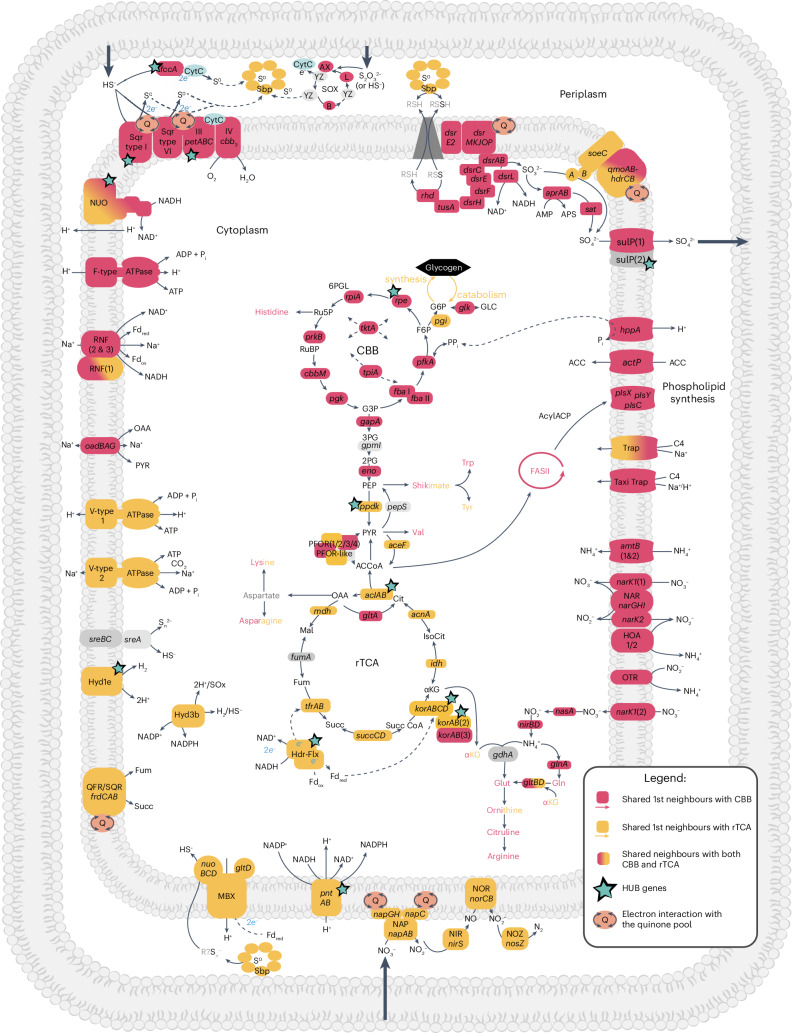
Co-expression patterns in Endoriftia carbon and energy metabolism. Gene colours indicate neighbours to the rTCA cycle (yellow) or CBB (red); stars mark hub genes. Genes/complexes of unknown function/direction in vivo: Hdr-Flx, Hyd1e, Hyd3b, MBX, HOA, OTR, QFR/SQR, RNF, OGOR/PFOR isoforms and *sreABC*. Metabolite abbreviations: 6PGL, 6-phosphogluconolactone; Ru5P, ribulose 5-phosphate; RuBP, ribulose-1,5-bisphosphate; G3P, glyceraldehyde 3-phosphate; F6P, fructose 6-phosphate; G6P, glucose 6-phosphate; GLC, glucose; 3PG, 3-phosphoglycerate; 2PG, 2-phosphoglycerate; PEP, phosphoenolpyruvate; PYR, pyruvate; AcCoA, acetyl coenzyme A; OAA, oxaloacetate; Cit, citrate; Mal, malate; Fum, fumarate; IsoCit, isocitrate; aKg, alpha-ketoglutarate; Suc, succinate; SucCoA, succinyl coenzyme A; Glut, glutamate; Gln, glutamine; Tyr, tyrosine; Trp, tryptophan; Val, valine. Illustration credit: Daria Chrobok, DC SciArt.

In the co-expression network, rTCA genes had 148 first neighbours, while CBB genes had 520, with only one shared between them (a gene of unknown function). Other metabolic systems shared first neighbours with either only the CBB or the rTCA. For example, genes associated with assimilatory nitrate reduction had first neighbours with the CBB, while those involved in dissimilatory nitrate reduction had first neighbours with the rTCA. The transmembrane bound nitrate reductase (Nar) and periplasmic nitrate reductase (Nap) can both be involved in assimilatory or dissimilatory nitrate reduction^23,24^. However, in the network, the narGHI genes shared first neighbours with genes that encode GOGAT (glutamate synthase, which is a key assimilatory enzyme), and the Nap genes shared first neighbours with *nosZ*, *norCB* and *nirS*, which function in the dissimilatory reduction of nitrite to N_2._ These data support a model whereby the Nar complex is operating in assimilatory nitrate reduction and the Nap complex is being used for dissimilatory nitrate reduction (Figs. 4a and 5). Most sulfur oxidation genes shared first neighbours with the CBB and not the rTCA (genes encoding Sox and rDSR, as well as *aprA*, *aprB*, *sat*, *sqrA* and *fccA*). An exception to this was seen in genes for sulfur globule proteins (Sgp) and genes for the membrane-bound sulfite-oxidizing enzyme (Soe), both first neighbours with the rTCA (Figs. 4b and 5). Both [NiFe] hydrogenases (1e and 3b), V-type ATPases, as well as a putative Mrp–Mbx complex (involved in zero valent sulfur (S^0^) reduction that is coupled to the production of a sodium motive force in other organisms^25^) shared first neighbours with the rTCA and not the CBB. Conversely, genes for cytochrome *bc*_1_ (encoded by *petABC*), a cytochrome *cbb*_*3*_ oxidase (COX) (complexes III and IV of the ETC), and F1-ATPase shared first neighbours with the CBB and not the rTCA (Figs. 4c and 5). The systems with genes that had first neighbours to both carbon fixation pathways are the Qmo (which is thought to transfer electrons from sulfite to the quinone pool)^26^, complex I of the ETC and some amino acid synthesis pathways (Figs. 4b and 5). However, when a system shared first neighbours with both, they had different genes (subunits) that are neighbours. In pathways for the biosynthesis of amino acids, the lysine, asparagine, ornithine, shikimate and phenylalanine/tyrosine pathways had genes with neighbours to both the CBB and the rTCA (Figs. 4d and 5). Endoriftia had genes encoding multiple isoforms of PFOR and OGOR, most of which shared first neighbours with either the rTCA or the CBB, which may offer a hint at in vivo function/direction (Fig. 5).

### Module–condition correlations

**Fig. 6:**
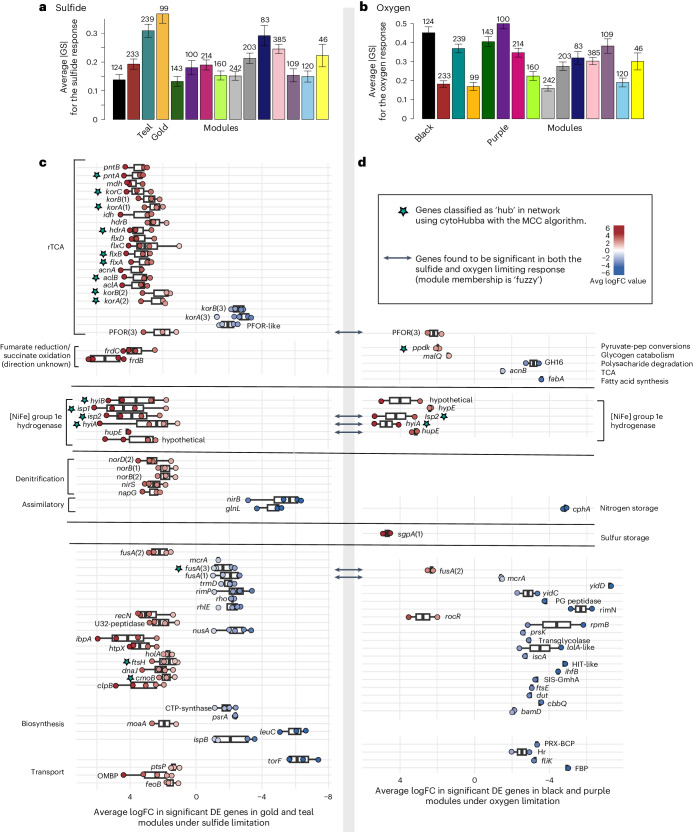
Gene expression patterns for genes most significantly associated with the sulfide or oxygen limitation response. **a**,**b**, Using Pearson’s correlation, the average absolute values of gene significance |GS| correlated with sulfide (**a**) and oxygen (**b**) across modules. **c**, DE patterns of the most sulfide-condition-associated genes in the gold and teal modules; red/blue dots indicate relative increase/decrease, with each dot representing a significant DE pairwise comparison. Error bars represent the standard deviation of the mean of all sulfide-limiting pairwise comparisons that showed significant DE (*P*_adj_ ≤ 0.05). **d**, DE patterns for the most oxygen-condition-associated genes within the black and purple modules; red/blue dots indicate relative increase/decrease. Error bars denote the standard deviation of the mean of all oxygen-limiting pairwise comparisons that showed significant DE. See Supplementary Tables 4–7 for mean values, standard deviation and *n* (number of comparisons used in the mean) for each gene found to be significant in these modules.

**Extended Data Fig. 6:**
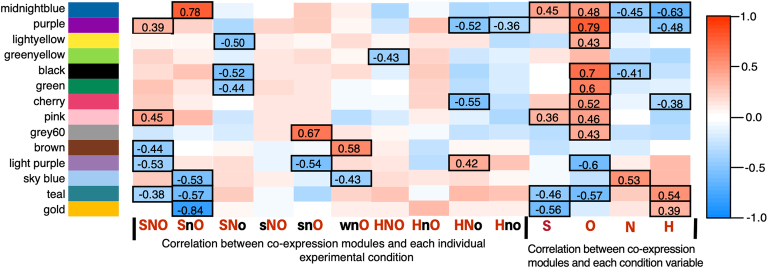
A module-condition heatmap showing the Pearson correlation between modules and aquaria conditions. A Pearson’s correlation coefficient was used to find the covariance between modules and aquaria (two-tailed) and p-values calculated using a student asymptotic test. Only correlation values that had a p-value of < 0.05 are shown. Correlation values on the left are for each aquaria condition. Correlation values on the right indicates a specific substrate (S^−^sulfide, O-oxygen, N-nitrate, H-hydrogen). For example, teal and gold modules are negatively correlated with sulfide replete conditions (S) indicating a pattern of an overall decrease in gene expression of these module’s eigengenes. Source data

The correlation between modules and vessel conditions was strongest with oxygen and sulfide (Extended Data Fig. 6). Modules gold and teal showed the highest correlation with dissolved ∑H_2_S concentrations, and purple and black with dissolved O_2_ concentrations (Fig. 6a,b). The most significant genes in the gold and teal modules, responding with an increase in DE under dissolved ∑H_2_S limitation, were genes encoding (1) rTCA (along with Hdr-Flx and *pntAB* in that gene cluster), (2) Hyd1e, (3) a membrane-bound quinol:fumarate oxidoreductase (encoded by *frdCAB*), which could also act as a succinate dehydrogenase (direction currently unknown), and (4) nitric oxide, nitrate and nitrite reductases (Nor, Nir and Nap). Conversely genes encoding a dimeric OGOR (*korAB*), a possible pyruvate ferredoxin oxidoreductase ‘PFOR-like’, a cytoplasmic nitrite reductase (*nirB*) and a nitrogen regulatory gene *glnL* all displayed a decrease in expression (Fig. 6c, and Supplementary Tables 4 and 5). The most significant genes within the black and purple modules, responding with an increase in DE, were genes encoding (1) Hyd1e, (2) enzymes involved in pyruvate conversions: a pyruvate phosphate dikinase (*ppdk*), a pyruvate ferredoxin oxidoreductase (PFOR(3), the number added to distinguish from other isoforms in the genome), (3) glycogen catabolism (*malQ*), and (4) a sulfur globule protein (*sgpA*). By contrast, a TCA cycle gene (*acnB*), a gene involved in polysaccharide degradation (GH16), and a nitrogen storage gene (*cphA*) showed a relative decrease in expression under O_2_ limitation (Fig. 6d and Supplementary Tables 6 and 7).

## Discussion

The discovery that a chemoautotrophic microorganism’s genome harbours the complete pathways for two disparate carbon fixation pathways led to many questions about the utility of having two such pathways^27^. Previous studies established that symbionts express both rTCA and CBB genes and proteins^7,28,29^, that individual symbiont cells are likely expressing both pathways, as well as spatial differences in expression within the worm host^30^.

We conducted extensive high-pressure incubations of live *Riftia* across a range of environmentally relevant geochemical conditions to assess the metatranscriptomic and metabolic responses of Endoriftia. This study directly measured carbon incorporation into the host and symbiont and revealed net carbon incorporation rates as high as 24 µmol g^−1^ h^−1^, rates that are higher than in most chemolithoautotrophic bacterial communities^31^, equal or greater than hydrothermal vent free-living microbial communities^32^, and on par with highly productive photosynthetic organisms^33–35^.

A co-expression network revealed a metabolic structure with the rTCA and CBB coupled to different and distinct metabolic processes. Notably, the CBB appears to be allied to (broadly speaking) aerobic processes, and rTCA allied to anaerobic processes. Of the 668 genes that share first neighbours with the CBB or the rTCA, only one is neighbours to both, suggesting regulatory independence. Notably, the co-expression patterns of many genes involved in energy transfer/electron flow, sulfide oxidation, and nitrogen metabolism are clearly linked to either the rTCA or the CBB cycle, but not both (Fig. 5).

Co-expression analysis identified genes likely to be the most significant and biologically relevant response for each environmental variable. rTCA genes and Hyd1e appeared to be important responses when carbon fixation was limited by reducing dissolved ∑H_2_S, with Hyd1e also significant with reduced dissolved O_2_, suggesting a prominent role when substrate concentrations limit energy availability for endosymbiont metabolism. Of note, sulfide oxidation and CBB genes were not found to be among these genes. Other genes that showed a significant increase in DE during substrate limitation include those encoding *frdCAB*, and PFOR(3). By contrast, there was a concomitant decrease in DE of PFOR-like and *korAB*(3), which may reflect a shift in isoform expression that could have an important role in maintaining metabolic function in different substrate regimes. Another important response to dissolved ∑H_2_S limitation appears to be an increase in DE of denitrification genes, along with a decrease in DE of genes involved in assimilatory nitrogen pathways.

While this study sheds light on the relationships between carbon, sulfide, oxygen and nitrogen metabolism, many aspects of the symbionts’ physiology are still unclear. Nowhere is this uncertainty more evident than in the role of hydrogenases in this symbiosis. A previous study^22^ showed that the provision of dissolved hydrogen does not support net carbon incorporation by the intact symbiosis. However, hydrogenase genes were expressed under all conditions, and all four subunits are ‘hub genes’ that appear to be closely connected to rTCA genes. These two [NiFe] hydrogenases (Hyd1e and Hyd3b) have been linked to sulfur metabolisms^36–38^. In many organisms, Hyd1e transfers electrons to polysulfide or sulfur reductases (encoded by *psrABC* and *sreABC*, respectively), resulting in ATP generation and H_2_S formation (or vice versa)^39–41^. The Hyd3b is a cytosolic NADPH-dependent and bidirectional enzyme with many putative functions: redox pool balance, fermentative H_2_ evolution via glycogen or pyruvate, and H_2_ or H_2_S evolution via S^0^ depending on conditions^42,43^. One possibility is that hydrogen is evolved under energy limiting conditions. This phenomenon is seen in cyanobacteria when they break down their glycogen stores during dark fermentation^44^. In addition, it is possible that internal hydrogen cycling is occurring (in which H_2_ is evolved by one hydrogenase and oxidized by the other), as seen in other organisms^45–47^.

The canonical genes for sulfide oxidation with oxygen, and genes involved in generating a proton motive force that drives ATP (*petABC*, and genes encoding CBB3 and F-type ATPase) all cluster with the CBB. This is consistent with the CBB cycle being the primary symbiont carbon fixation pathway; however, *δ*^13^C evidence indicates a mixed carbon fixation strategy, and genes for both pathways are highly expressed (this study and refs. ^7,28,29^). We also see genes for the rTCA, the FBEB Hdr-Flx complex, a Hyd1e and a transhydrogenase appearing as hub genes in our network analysis, which underscores the importance of these systems in Endoriftia.

The role of oxygen in this symbiosis also warrants further exploration. We know that large quantities of oxygen are consumed by the host and symbiont when dissolved sulfide is abundant (this study and refs. ^20,21,48^). Yet, we do not know the dissolved oxygen regime inside the trophosome, where microoxic conditions could occur due to the presence of high-affinity haemoglobins and oxygen demand. Because the rTCA relies on oxygen-sensitive Fd_red_, and we see the complete denitrification pathway clustering with the rTCA, this study supports the idea that the rTCA is active in parts of the trophosome lobules with lower O_2_. These data point to a partitioned metabolism where sulfide oxidation and carbon fixation occur via the CBB cycle, while the rTCA cycle is active and concurrently fixing carbon, with modulations of expression occurring under differing redox regimes. This strategy may enable the host–symbiont association to have high productivity rates and maintain autotrophic poise in the stochastic vent environment.

Finally, modularity in co-expression networks, such as seen in our data, is thought to emerge in organisms that encounter environmental stressors, where this reorganization of the network may increase robustness to perturbation^19^. In fact, some of the most central genes in the network related to substrate limitation were genes of the rTCA cycle and the Hyd1e, underscoring their importance in supporting metabolism during periods of redox stress. Further experimental studies are needed to understand what these roles may be. Nevertheless, we posit that this mode of autotrophy may represent a new carbon fixation modality that confers a fitness advantage in a highly dynamic environment where redox conditions are continuously changing. Continued investigation of these processes could provide deeper insights into the evolution of carbon fixation pathways, as well as the evolutionary histories of microorganisms that harbour multiple pathways^20^.

## Methods

### Research expeditions, study sites and *Riftia* collections

The data presented here were generated using *Riftia* collected during two research cruises on board the RV *Atlantis*, the first in November 2014 (AT26-23) and the second in October 2016 (AT37-04). Collections took place during dives with the Human Occupied Vehicle (HOV) *Alvin* at the East Pacific Rise (EPR) vent sites ‘Crab Spa’, ‘Tica’ and ‘Bio9’, all of which are areas of active basalt-hosted hydrothermal vents located near 9° 50’ N, 104° 18’ W at ~2,500 m water depth. These sites are characterized by hydrothermal fluid having elevated ∑DIC, ∑H_2_S, modest concentrations of dissolved NO_3_^−^, very little NH_4_^+^ and variable amounts of H_2_, surrounded by well-oxygenated bottom water^29,49^. For the experiments, small to moderately sized *Riftia* (≤30 cm in length) were collected towards the end of each dive to minimize their time in non-vent conditions, and brought to the surface via a thermally insulated container on the submersible.

### Replicating the *Riftia* in situ energetic landscape

Upon recovery, *Riftia* that were responsive to touch were quickly placed into high-pressure aquaria within the high-pressure respirometry system (HPRS).

The HPRS includes four acrylic gas equilibration columns that are filled with 0.2-µm-filtered seawater then bubbled with select gases (H_2_, H_2_S, CO_2_, O_2_, with N_2_) to re-create vent-like fluids. This fluid feeds the custom-built 2.5 l titanium aquaria via high-pressure pumps (Lewa America) where pressure is maintained at ~20 MPa via back-pressure relief valves (Staval). Flow rates are maintained at ~50 ml min^−1^. For biological replication, 4–6 tubeworms were placed into each high-pressure aquarium per treatment. Worms were given 12–24 h to acclimate in aquaria before experimental conditions were started. To simulate the observed differences in hydrothermal fluid composition, different treatments were run in which we incubated worms in 0.2-μm-filtered sterilized seawater with various dissolved concentrations of ∑H_2_S, O_2_, NO_3_^−^ and H_2_ (Fig. 1d,e and Supplementary Table 1). These treatments can be generalized into (1) ‘replete’ conditions, in which ∑H_2_S, O_2_ and sometimes NO_3_^−^ were abundant in the aquaria seawater; (2) ‘limiting’ conditions, in which one or more of these substrates were deficient or nearly absent in the aquaria seawater; and (3) ‘controls’, in which the animals were incubated without any substrate amendments to the seawater to simulate the cessation of venting (conditions are shown in Supplementary Table 1). Across all these treatments, pH and ∑DIC were held steady at ~6.5 and 4–6 mM, respectively. Worms were maintained in the aquaria at experimental conditions for 2–3 days. It is worth noting that since multiple *Riftia* were incubated in each vessel, the treatments were not applied independently, introducing a possible spillover effect. Given the difficulty in working with deep-sea organisms, as well as the lifestyle of these worms, we felt that this was the best method because (1) limited time at sea meant it was impractical to incubate 30 worms in separate aquaria; (2) aquaria seawater conditions were effectively steady state, thus minimizing spillover effects due to geochemistry; and (3) *Riftia* is found in very tightly packed clumps, and we believe that putting them together more closely mimics in situ conditions. Finally, this study focuses on the symbiont population within each *Riftia*, wherein each worm already governs the conditions around the symbionts.

To measure the consumption of dissolved gases by the tubeworms during the experimental treatments, fluid was collected before entering the aquaria (incurrent) and upon exiting the aquaria (excurrent). Dissolved H_2_S was measured by collecting and preserving incurrent and excurrent fluid subsamples with 2 mM zinc acetate solution for subsequent analyses using a colorimetric sulfide quantification assay (LaMotte, with absorbance read at 670 nm on a Spectramax i3 plate reader). Dissolved H_2_ was measured with a Unisense H_2_ minisensor 500 flow cell (range = 0–800 µM; detection limit 0.3 µM) and dissolved O_2_ was measured with a Presens oxygen FTC-SU-PSt3-S flow cell (range = 0–1,400 µM) by placing both sensors in line with the incurrent and excurrent fluid lines.

During the 2014 cruise, ∑H_2_S was consistently measured, whereas during the 2016 cruise, O_2_ and H_2_ were also measured as described above. Upon termination of the experiments, the aquaria were depressurized and *Riftia* were weighed on a motion-compensated shipboard balance^50^, dissected at 4 °C, and sampled for metatranscriptomic sequencing and isotope analyses (described in the sections below). Uptake rates of ∑H_2_S, O_2_ and H_2_ were calculated as (intake − outtake)/(total biomass in aquaria × hour)^51^. To compare uptake rates among different treatments, a generalized least square model in R was used that accounted for unequal variances (as seen between high and low amounts of substrates) and allowed for dependence between timepoints that were closer together. The least square (LS) means of these uptake rates were compared using a pairwise-adjusted Holmes test (two-sided), with an *α* value of 0.05.

### Determining individual tubeworm carbon fixation rates

To robustly determine the rate of inorganic carbon fixation and incorporation into biomass during experimental treatments, a stock solution of 99% NaH^13^CO_3_ was added to the intake seawater reservoir to achieve a final isotopic abundance of 2.64% ^13^C of the ∑DIC. During the course of the experiments, multiple intake and outtake fluid samples were collected and filtered using 0.2 μm disposable filter capsules, then stored in vacuum-evacuated Exetainers (Labco) for later isotopic analysis where stable isotope ratios in each sample were measured using a Deltaplus XP mass spectrometer at the Yale Analytic and Stable Isotope Center (YASIC).

After cessation of experiments, symbiont-containing tissues (the trophosome) and non-symbiont host tissues (gill and skin) from each worm were frozen at −80 °C for later isotopic analyses (Fig. 1f). In the laboratory, the tissues were lyophilised using a FreeZone 2.5 freeze dryer (Labconco) to minimize carryover of inorganic carbon; all lyophilised samples were bathed in 0.1 N HCl for ~10 min, rinsed in deionized water and then dehydrated in a vacuum oven (Labconco) at 50 °C. Once dried, the tissues were finely ground using a glass mortar and pestle and dispatched to the Boston University Stable Isotope Laboratory. There, samples were placed into tin capsules and precisely weighed using a microelectronic balance, and then combusted in a carbon and nitrogen analyser (Eurovector). The resultant gases were separated by chromatography and analysed using a GV Instruments IsoPrime isotope ratio mass spectrometer. The ratios of ^13^C to ^12^C isotopes were determined against international standards such as NBS 20 (Solenhofen Limestone), NBS 21 (spectrographic graphite) and NBS 22 (hydrocarbon oil). The ∂^13^CV-PDB values (per mille) were calculated using equation (1), with *R* being the atomic ratio of ^13^C/^12^C.

1 $${\partial }^{13}C=\left(\frac{{R}_{{\rm{sample}}}}{{R}_{{\rm{standard}}}}-1\right)\times 1,000$$

The method used to calculate the incorporation rates of DIC into tissues was adapted from ref. ^51^. This involved computing the atomic percentages (*A*%) for both labelled and naturally abundant samples, using their *δ*^13^C values such that:

2 $$A \% =\left(\frac{{R}_{{\rm{sample}}}}{{R}_{{\rm{sample}}}+1}\right)\times 100$$

To calculate the percentage of ^13^C that was incorporated (%^13^*C*_inc_) into biomass during the course of the experiments, the *A*% of the tissue from worms that were not exposed to the isotopic label (*A*%_nat_) was subtracted from the *A*% of tissue from experimental worms exposed to labelled inorganic ^13^C (*A*%_lab_), and this was divided by the *A*% of DI^13^C of the fluid (*A*%_wat_) the experimental worms were incubated in (for example, how much isotopic label they were exposed to) subtracted by the *A*%_nat_ (see below):

3 $$\% {{13\atop}C}_{{\rm{inc}}}=\left(\frac{{A \% }_{{\rm{lab}}}-{A \% }_{{\rm{nat}}}}{{A \% }_{{\rm{wat}}}-{A \% }_{{\rm{nat}}}}\right)$$

The total weight of ^13^C incorporated (*W*^13^*C*_inc_) was calculated by multiplying the $\%^{13}C_{{\rm{inc}}}$ by the total dry weight of the tissue analysed, and this value was used to calculate the rate of carbon incorporated per dry weight amount (Dry*C*_inc_) expressed as µM ^13^C g^−1^ h^−1^, where MW stands for the molecular weight of ^13^C:

4 $${{{\rm{Dry}}C}}_{{\rm{inc}}}=\frac{\left[\left({W}^{\,13}{C}_{{\rm{inc}}}/{\rm{MW}}\right)\times 1,000\right]}{\left({\rm{DW}}\times {\rm{hours}}\right)}$$

This Dry*C*_inc_ was converted into a wet weight *C*_inc_ rate by multiplying this value by the dry weight (DW) to wet weight ratio for each sample. The *C*_inc_ values reported in this paper were from the symbiont-bearing tissue (the trophosome). However, the plume and the skin were also analysed. For each tissue type, the *A*%_nat_ value was calculated by using the mean values for the natural abundance of these tissue types.

The trophosome tissue is ~24% symbionts by volume^52^ and is the vascularized organ that contains the specialized host cells that house the symbionts. The ^13^C incorporation rates herein represent the net carbon fixation attributable to symbionts’ sulfide-dependent chemoautotrophic carbon fixation. Although there is evidence that the tubeworm can carboxylate pyruvate to a 4-carbon organic acid (such as succinate or malate)^53,54^, those rates are insufficient to support net growth. Moreover, they would not likely be stimulated by the provision of sulfide because only the symbionts can use that as an electron donor. Thus, any carbon incorporation due to host carboxylation reactions is represented by the ^13^C incorporation rates measured in the absence of sulfide (that is, the ‘no sulfide’ conditions), which also does not exclude the occurrence of symbiont autotrophy utilizing elemental sulfur stores under these conditions.

The rate of net inorganic carbon incorporated into biomass (*C*_inc_) was calculated as micromoles per gram wet trophosome weight per hour (µmol g^−1^ h^−1^). Rates were compared in R using a linear mixed effects model that accounts for unequal number of *Riftia* per treatment, with *C*_inc_ as the dependent variable, aquaria condition and sample replicate as fixed effects, with each worm being a random effect: lmer(*C*_inc_ ≈ aquaria conditions + sample_rep + (1| wormID)). This model was used to calculate the LS means of *C*_inc_. LS means were compared using a pairwise-adjusted Holmes test (two-sided), with an *α* value of 0.05.

### Symbiont RNA extraction and sequencing

Messenger RNA was sampled from the trophosome tissue, which contains symbionts, of 30 separate worms. For each condition in the aquarium, three worms were used as biological replicates. To quickly stabilize mRNA, symbiont-containing trophosome tissues were immediately homogenized with a Tissue-Tearor homogenizer (BioSpec) in 1 ml of TRIzol reagent (Thermo Fisher), or placed in 5 ml of RNALater (Thermo Fisher), allowed to incubate for ~8 h at 4 °C and then stored at −80 °C. For both RNALater and TRIzol stored samples, total RNA was extracted using the Direct-zol RNA MiniPrep kit (Zymo Research), following manufacturer instructions. Extracted RNA quality was checked using an Agilent Bioanalyzer 2100. Total RNA was normalized and sent to the Microbial ’Omics Core (MOC) at the Broad Institute (Cambridge, Massachusetts), where DNA and ribosomal RNA removal, library prep and sequencing were completed. Complementary DNA libraries were constructed from 0.5 to 1 µg of RNA using a modified RNAtag-seq protocol^55^, whereby an adaptor was added to the 3’ end of the cDNA by template switching using SMARTScribe (Clontech) after reverse transcription^56^. cDNA was sequenced with a 2 ×33- to 75-bp paired-end protocol using the Illumina Novaseq 6000 platform.

### Illumina sequence read preprocessing

The quality of raw, unfiltered sequencing reads was assessed using FastQC (v.0118) (https://www.bioinformatics.babraham.ac.uk/projects/fastqc/). This assessment confirmed that, while no aberrant base quality-by-cycle profiles were detected, there was some evidence for retained adaptor sequence, as well as an abundance of over-represented sequences. The latter often reflect undesirable enrichments for non-target sequences such as rRNAs or other ubiquitous non-coding sequences. To the extent that these reads map to annotated transcripts, these reads impact normalization methods and provide an over-optimistic picture of statistical power for downstream differential expression analyses. Thus, reads were subsequently processed to eliminate these biases. First, adaptor sequences were removed with TrimGalore! (v.0.6.5) (www.bioinformatics.babraham.ac.uk/projects/trim_galore/), setting the minimum retained read length, stringency and error rate to 35, 5 and 0.01, respectively. Trimming low quality bases from reads was not undertaken because (1) base qualities of libraries was high overall and (2) quality trimming of reads has been shown to distort expression estimates^57^. Second, over-represented reads were removed (https://github.com/harvardinformatics/TranscriptomeAssemblyTools). Finally, we filtered out reads originating from rRNAs by mapping read pairs to the SILVA rRNA database^57^ (release 138) and removing read pairs for which ≥1 read aligned to the database. Specifically, sequences for SSURef NR99 were transformed to DNA space by replacing uracil (U) bases to thymine (T). Reads were then aligned to the database with Bowtie2 (v.2.5.3)^58^ in ‘very-sensitive-local’ mode. Sequencing the combined host/symbiont trophosome tissue using Illumina Novaseq yielded an average of 31 M paired-end reads per sample. After the removal of rRNA and over-represented reads, there were an average of 19 M remaining reads. The percentage of these reads that mapped to the symbiont genome averaged 2.6 M (14%), which is sufficient to detect differential expression with statistical significance^59^. Of the 3,316 genes in the published genome, 3,140 (~94.6%) were sufficiently abundant (defined as greater than one count per million in three or more samples) to be included in the analyses.

### DE analysis

Transcript abundances were first quantified with RSEM^60^ (v.1.3.1) against the recently published, complete reference genome for ‘*Ca*. Endoriftia persephone’ (RefSeq accession GCF_023733635.1)^61^. DE between specific pairs of conditions was carried out in a linear modelling framework with the limma-voom (v.3.17) package in R (v.4.0–4.3.3)^62,63^. In a comparison of multiple differential expression tools, limma-voom was a top performer along with sleuth^64^. Within limma-voom, expression estimates were normalized using the TMM method^65^, and only genes that showed expression values greater than one CPM in three or more libraries were used before DE testing. Because an initial principal component analysis (PCA) of expression data indicated that some samples appeared to be outliers and did not cluster with other samples derived from the same condition, we took advantage of a unique feature of limma-voom by estimating ‘precision weights’ for each sample and incorporating these weights into DE testing. These precision weights account for variance between different observations, so that poor-quality samples will be ‘down-weighted’ in the analysis. Furthermore, this approach precludes the necessity of discarding these samples, which is particularly an issue with bulk RNA-seq experiments for which biological replication is typically low. DE tests were performed by first fitting a linear model, via a design matrix, to the entire dataset. Comparisons between pairs of conditions of interest were then performed by extracting linear contrasts for these comparisons, followed by empirical Bayes moderation of the standard errors. Differentially expressed genes were determined using a false discovery rate (FDR) cut-off of ≤0.05, which was calculated using the Benjamini–Hochberg method, a standard approach for FDR calculations in DE testing frameworks. We note that gene expression data are not a quantitative representation of carbon fixation rates, and transcription does not always correlate with protein abundances; however, previous studies have found a much tighter correlation when looking at population-level abundances after environmental steady states have been reached (as in this study)^66^.

### Gene annotation

To identify the function of genes that have unclear annotations, we deployed a variety of approaches using these programmes: HydDB (https://services.bire.au.dk/hyddb/), DeepTMHMM (https://dtu.biolib.com/DeepTMHMM), HMMR (https://www.ebi.ac.uk/Tools/hmmer/), STRING (https://string-db.org/), Metal Predator (http://metalweb.cerm.unifi.it/tools/metalpredator/), NCBI BLAST, NCBI COBALT and CD search (www.ncbi.nlm.nih.gov/home/analyze/).

### Weighted gene co-expression network analysis

Co-expression patterns were analysed using the Weighted Gene Co-Expression Network Analysis (WGCNA) package (v.1.72) in R^67^. This analysis was unsupervised, meaning no previous filtering according to DE or function was performed. Of the 3,140 genes that were used in the DE analysis above, only the top 2,500 genes that displayed the highest variable expression were used.

Standard methods from the WGCNA pipeline were used to construct a co-expression signed hybrid network, with the normalized and weighted gene expression profiles from limma-voom as input and using refs. ^67–69^. Analysis of the fit index and mean connectivity revealed that a soft threshold power of *β* = 8, for a signed hybrid network, adhered to a scale-free topology model the best, in line with biological assumptions. Using this soft thresholding power of 8, genes were clustered into 14 modules after merging highly connected modules. An analysis of co-expression patterns in individual samples with aquaria condition and cruise year was visualized with the sample dendrogram aquaria condition heat map, which shows no apparent outliers and no discernible batch effect by cruise year. The resulting network was visualized and further analysed in Cytoscape (3.9.1)^70^. Before import into Cytoscape, these genes were filtered according to edge weight >0.05, which left 1,945 genes. In Cytoscape, the network was visualized using the perfuse force directed layout. Network scoring to find hub genes was done using cyto-Hubba (v.0.1), a Cytoscape plugin that performs hub object analysis^71^ using the maximal clique centrality (MCC) method, as suggested by the authors. Cytoscape was also used to find the nearest neighbours of genes, which were manually grouped according to putative metabolic function. From these metabolic groups, the intersection between sets of first neighbours was visualized using the R programme ‘UpSetR’ (v.1.40)^72^.

To identify genes that are biologically relevant to the phenotypic response to sulfide and/or oxygen limitation, we used an approach commonly utilized in human disease research to search for candidate drug targets and/or mechanisms^73–76^. This method relates network structure (modules) to external conditions or traits by calculating (1) gene significance (GS), which is the Pearson correlation between a given gene and the external condition variable (that is, sulfide and/or oxygen), and (2) module membership (MM), which is the correlation between each gene and the module eigengene (ME) representing the first principal component of each module. For each condition variable, we looked at the most significant genes within the two most significant modules (calculated by having the highest average gene significance for that variable). The condition variables were assigned binary values for each condition, such that an assignment of one was given to treatments that were replete with sulfide (or oxygen), and zero to treatments that were limiting in that substrate. Genes were classified as significant for that condition variable if their |GS| > 0.2, with a *P* ≤ 0.05 and their |MM| > 0.8. These genes were further filtered on the basis of the limma results for DE for that condition variable. Since the limma DE analysis was calculated by comparing the DE of one treatment against another, there were five sulfide and two oxygen limitation comparisons used to calculate the average logFC for each condition variable (Supplementary Tables 4–7).

### Statistics and reproducibility

*Riftia* used in these experiments were selected on the basis of size (due to size constraints of the aquaria) and responsiveness. After this non-random sampling, *Riftia* were randomly assigned to treatment aquaria. *Riftia* that died during the course of the experiments were excluded from analyses. No other data were excluded from analyses. No statistical method was used to predetermine sample size in aquaria. The investigators were not blinded to allocation during experiments and outcome assessment.

### Reporting summary

Further information on research design is available in the Nature Portfolio Reporting Summary linked to this article.

## Supplementary information

Supplementary Information Supplementary Tables 1–7.

Reporting Summary

Peer Review File

## Source data

Source Data Fig. 2 Intake and uptake rates for each timepoint (Fig. 2a); a list of genes in each functional category, their DE expression patterns and the functional frequency table used to make Fig. 2b (source data for Fig. 2c not provided as this is included in source data for 2b; one can find each DE comparison used for these genes within the table provided).

Source Data Fig. 4 Binary files for each gene for each functional category used in Fig. 4a–d, with zero indicating no shared first neighbours and 1 indicating shared first neighbours in the WGCNA network. These are the files used to make the figures in UpsetR.

Source Data Extended Data Fig. 2 DE expression patterns and the COG frequency table used to make the figure.

Source Data Extended Data Fig. 6 A list of each gene in each COG category, their DE expression patterns and the frequency table used to make the figure.

## Data Availability

Raw sequencing data have been submitted to the NCBI Sequence Read Archive (SRA: SRP323622; project ID: PRJNA736714). Processed data files (read counts, differential expression and co-expression analyses) have been deposited in the NCBI Gene Expression Omnibus (GEO) and are accessible through GEO Series accession number GSE249345. All other data are available in the supplementary material. Source data are provided with this paper.

## Code availability

All code used in these analyses are available in GitHub at https://github.com/harvardinformatics/EndoriftiaTranscriptomics or via Zenodo at 10.5281/zenodo.10894444 (ref. ^77^).
