# Supplementary material for: Co-expression analysis reveals distinct alliances around two carbon fixation pathways in hydrothermal vent symbionts
Source: Nat Microbiol. 2024 Jun 5;9(6):1526–39. doi: 10.1038/s41564-024-01704-y (PMC11636981; doi:10.1038/s41564-024-01704-y)
Supplement: Supplementary file 1 — Supplementary Tables 1–7. [file 41564_2024_1704_MOESM1_ESM.pdf]

nature microbiology

Supplementary information

https://doi.org/10.1038/s41564-024-01704-y

Co-expression analysis reveals distinct alliances around two carbon fixation pathways in hydrothermal vent symbionts

In the format provided by the authors and unedited

Supplementary Table 1. Aquaria conditions and uptake rate measurements (mean and standard error reported) for each experiment conducted during the EPR2014 and EPR2016 expeditions.

An na indicates that the measurements were not taken, due to the substrate not being present. A dash indicates the measurements were not taken due to lack of instrument availability or mortality in aquaria that would render uptake rates inaccurate. Condition abbreviations: S-sulfide replete; s-sulfide present but limiting, H-hydrogen replete, N-nitrate replete, **n**-nitrate limiting, O-oxygen replete, **o**-oxygen limiting; **w**-sea water which indicates a no vent amended condition (pH ~8.0, high O_2, no NO_3^-, DIC ~ 2mM) similar to bottom water.

	**Aquaria conditions intake values (µM)**				**Substrate uptake LS means (µmol g-1 h-1)**			**Experimental parameters**			
**Condition abbreviation**	**H_2**	**$\sum H_2S$**	**O_2**	**NO_3^-**	**$\sum H_2S$**	**O_2**	**C_{inc}**	**Worms (n)**	**Biomass (g)**	**Time (hours)**	**Year**
**SnO**	na	215 ± 59 (n =15)	366 (n=2)	na	13.5 ± 1.1 (n=15)	-	5.4 ± 0.5 (n=4)	4	36.3	65	2014
**snO**	na	51 ± 10 (n=17)	391 (n=1)	na	1.3 ± 0.1 (n=17)	-	1.9 ± 0.4 (n=5)	5	137.1	70	2014
**HnO**	43 ± 1 (n=2)	na	380 (n=1)	na	-	-	2.2 ± 0.05 (n=4)	4	-	30	2014
**wnO**	na	na	~380*	na	na	-	1.0 ± 0.05 (n=4)	4	61.4	68	2014
**SNO**	na	154 ± 33 (n=13)	292 ± 11 (n=13)	40*	8.2 ± 3.0 (n=13)	11.0 ± 0.8 (n=13)	16.4 ± 1.9 (n=4)	6	48.14	52	2016
**SNo**	na	160 ± 43 (n=13)	32 ± 8 (n=13)	40*	2.7 ± 2.0 (n=13)	1.0 ± 0.1 (n=13)	0.7 ± 2.7 (n=2)	4	66.7	52	2016
**sNO**	na	29 ± 0.0 (n=18)	315 ± 38 (n=18)	40*	-	-	-	3	-	70	2016
**HNO**	114 ± 5 (n=15)	na	271 ± 42 (n=15)	40*	na	2.3 ± 0.3 (n=15)	0.7 ± 1.9 (n=4)	5	-	60.3	2016
**HNo**	113 ± 1 (n=14)	na	31 ± 6 (n=14)	40*	na	0.3 ± 0.0 (n=14)	0.2 ±1.9 (n=4)	5	222	60.3	2016
**Hno**	72.3 ± 5 (n=14)	na	13.4 ± 3 (n=15)	40*	na	-	0.3 ± 1.9 (n=4)	5	228	60.3	2016

*No direct measurements available, estimates based on how much gas was bubbled into the column (for oxygen), or how much sodium nitrate was added to intake tank

Supplementary Table 2: Summary of test statistics from the least squares (LS) mean comparisons of sulfide and oxygen uptake rates in aquaria, and carbon incorporation rates in individual worms.

Contrast comparisons are denoted by abbreviations explained in Supplementary Table 1, with variables in parentheses representing the identical conditions between aquaria. In R a linear mixed effect model (lmer) was used with C_{inc} as the dependent variable, aquaria condition and sample rep as fixed effects with each worm being a random effect; lmer(C_{inc} ~ aquaria conditions + sample_rep + (1| wormID)). For sulfide and oxygen uptake rates, in R a linear model using generalized least squares which takes into account that observations will be more correlated between sampling time points; gls(uptake ~ aquaria conditions, weights = varIdent(form = ~ 1 | group), correlation = corAR1(form = ~ hours | ID)). Least square means from these models were contrasted using a pairwise adjusted Holm method. Significantly different LS means are highlighted in bold. Refer to Supplementary Table 1 for aquaria conditions and descriptive statistics.

Year	Variable	Contrast	Estimate	SE	df	t.ratio	p.value
**2014**	**Sulfide**	**$s_{vs}S$ (nO)**	**-12.278**	**1.096**	**28.000**	**-11.204**	**0.000**
**2016**	**Sulfide**	**$o_{vs}O$ (NO)**	**-5.562**	**1.030**	**22.000**	**-5.402**	**0.000**
**2016**	**Oxygen**	**$o_{vs}O$ (HN)**	**-2.067**	**0.315**	**46.000**	**-6.560**	**0.000**
**2016**	**Oxygen**	**$H_{vs}S$ (No)**	**-0.758**	**0.123**	**46.000**	**-6.158**	**0.000**
**2016**	**Oxygen**	**$H_{vs}S$ (NO)**	**-8.666**	**0.885**	**46.000**	**-9.790**	**0.000**
**2016**	**Oxygen**	**$o_{vs}O$ (SN)**	**-9.975**	**0.836**	**46.000**	**-11.927**	**0.000**
**2014**	**C_{inc}**	**$H_{vs}S$ (nO)**	**-3.269**	**0.708**	**13.000**	**-4.615**	**0.002**
2014	C_{inc}	$H_{vs}s$ (nO)	0.291	0.672	12.998	0.433	0.672
2014	C_{inc}	$H_{vs}w$ (nO)	1.209	0.708	13.000	1.707	0.335
**2014**	**C_{inc}**	**$s_{vs}S$ (nO)**	**3.560**	**0.672**	**12.998**	**5.298**	**0.001**
**2014**	**C_{inc}**	**$w_{vs}S$ (nO)**	**4.479**	**0.708**	**13.000**	**6.323**	**0.000**
2014	C_{inc}	$w_{vs}s$ (nO)	0.918	0.672	12.998	1.367	0.390
2016	C_{inc}	$o_{vs}O$ (HN)	0.458	2.677	13.000	0.171	1.000
**2016**	**C_{inc}**	**$H_{vs}S$ (NO)**	**-15.770**	**2.677**	**13.000**	**-5.890**	**0.000**
2016	C_{inc}	$N_{vs}n$ (Ho)	-0.132	2.677	13.000	-0.049	1.000
2016	C_{inc}	$H_{vs}S$ (No)	-0.484	3.279	13.000	-0.148	1.000
**2016**	**C_{inc}**	**$o_{vs}O$ (SN)**	**15.744**	**3.279**	**13.000**	**4.801**	**0.002**

Supplementary Table 3: A list of the top 50 hub genes, ranked by Maximal Clique Centrality (MCC) in cytoHubba.

Listed in order of highest MCC values and decreasing. Modules listed are the modules that these genes have the highest module membership (MM) in, calculated in WGCNA. Only broad functions of genes discussed in this paper are listed for clarity.

NCBI Accession	Broad function	Gene/product	Module	MCC	Notes
WP_005962037.1		PPIase	pink	63	
WP_039960513.1	CBB?	Thioredoxin	cherry	62	upstream of CBB genes
WP_040820191.1		Ctc	cherry	62	
WP_005965298.1		*fusA*(2)	pink	60	
WP_005960941.1		*rpoA*	pink	57	
WP_039960560.1	sulfide oxidation	SqrA	pink	56	sulfide:quinone oxidoreductase Type 1
WP_006475076.1		IscA	cherry	55	
WP_005965407.1		*rimM*	pink	52	
WP_005960739.1	hydrogenase	Isp1	teal	51	[NiFe] Group 1e
WP_006475237.1		*purF*	pink	49	
WP_005962937.1		*arsS*	brown	42	
WP_040819914.1	hydrogenase	HyiB	teal	42	[NiFe] Group 1e
WP_006473784.1	carbon metabolism	*ppdk*	teal	40	pep_pyruvate_oxalalactetate node
WP_005965033.1		*nusA*	pink	40	
WP_006475471.1	hydrogenase	HyiA	teal	37	[NiFe] Group 1e
WP_005963126.1	sulfide oxidation	FccA	cherry	37	Flavocytochrome c sulfide dehydrogenase
WP_050799361.1		XylB	cherry	36	
WP_005958750.1		OmpR	brown	36	
WP_005965046.1		*truB*	pink	36	
WP_040819861.1	rTCA	AclB	gold	35	ATP-citrate lyase, beta subunit
WP_006473707.1		*cmoB*	teal	34	
WP_006475793.1		*rplJ*	pink	34	
WP_006474199.1		AtoC	brown	32	
WP_005961639.1		RND_mfp	brown	32	
WP_005963052.1		RpoD	brown	32	
WP_005959134.1	CBB	*rpe*	cherry	29	also involved in oxidative PPP
WP_005960944.1		*rpsM*	green	29	
WP_005965507.1	rTCA	HdrA	gold	27	Hdr-Flx in putative rTCA operon
WP_005958557.1		*ribF*	brown	27	
WP_006475120.1		*hemA*	cherry	26	
WP_039960060.1	rTCA	KorA(2)	teal	26	OGOR (not in main rTCA operon)
WP_006473547.1		PQQ_DH_like	brown	24	
WP_006475472.1	hydrogenase	Isp2	teal	23	[NiFe] Group 1e
WP_040819878.1	rTCA	PntA	gold	19	transhydrogenase in putative rTCA operon
WP_005965504.1	rTCA	FlxB	gold	18	Hdr-Flx FBEB on putative rTCA operon
WP_005958751.1	Sulfate transporter	SulP(2)	brown	17	
WP_006475064.1	rTCA	KorB(2)	teal	16	OGOR (not in main rTCA operon)
WP_006475148.1	biosynthesis	*asnB(2)*	cherry	15	
WP_005959845.1		*rng*	brown	14	
WP_006475002.1		*uvrD*	lightpurple	14	
WP_005960985.1		*bamB*	brown	13	
WP_251859198.1	rTCA	FlxA	gold	11	Hdr-Flx FBEB in putative rTCA operon
WP_005965508.1	rTCA	KorC	gold	11	OGOR in putative rTCA operon
WP_005961932.1	ETC	PetB	pink	10	cytochrome bc_1 (complex III)
WP_006474511.1		Theoredoxin_like	brown	9	
WP_005959787.1		hypothetical	teal	9	
WP_006474944.1	ETC	*nuoL*	sky blue	8	NADH dehydrogenase (complex I)
WP_240991664.1		*ftsH*	teal	7	
WP_006474335.1		ABC_transporter (MlaF)	brown	6	
WP_006475391.1	rTCA	KorA(1)	gold	5	OGOR on rTCA operon

Supplementary Table 4: Genes significant for sulfide condition in gold module.

Key genes in the gold module linked to sulfide response. Selection criteria include genes with |GS| > 0.2 and Pearson p-value < 0.05, along with | MM | > 0.8, both determined using Pearson correlations with two-tailed Student's p-values.   These were further filtered for genes with significant differential expression (DE) with adjusted p-value (padj) < 0.05, determined via a robust two-sided linear model (limma package) with Benjamini-Hochberg adjustment for multiple comparisons. All of the genes that met the first parameter, were also significantly DE in one of these comparisons. The average of these significant DE comparisons is reported below as mean logFC, with standard deviation and number of comparisons in parentheses. The sulfide comparisons where these genes were DE and thus used in these averages were: $w_{vs}S(nO)$, $s_{vs}S(nO)$, $H_{vs}S(NO)$, and $H_{vs}S(nO)$.

Gene/product	mean logFC	Functional category	Accession	Locus ID
glnL- nitrogen regulation protein NR(II)	-4.58 (0.73,3)	nitrogen regulatory	WP_006474975.1	L0Y14_RS00080
nirB- nitrite reductase large subunit NirB	-5.22 (1.43,4)	assimilatory nitrite reduction	WP_006473537.1	L0Y14_RS01985
frdB- SQOR family type B, subunit B	5.48 (1.11,4)	succinate:quinone oxidoreductase	WP_005965241.1	L0Y14_RS07695
U32_peptidase	2.05 (0.78,4)	protein regulation	WP_240991716.1	L0Y14_RS10315
leuC- 3-isopropylmalate dehydratase large subunit	-5.97 (0.74,3)	amino acid biosynthesis	WP_005961310.1	L0Y14_RS10410
recN- DNA repair protein	2.70 (0.99,4)	DNA repair	WP_006473937.1	L0Y14_RS11255
HdrB- CoB--CoM heterodisulfide reductase iron-sulfur subunit B	2.44 (0.59,3)	Hdr-Flx- in putative rTCA operon	WP_040819862.1	L0Y14_RS11965
KorA(1)- 2-oxoacid:acceptor oxidoreductase subunit alpha	2.50 (0.57,3)	C-Fixation - in putative rTCA operon	WP_006475391.1	L0Y14_RS11975
KorB(1) - 2-oxoacid:ferredoxin oxidoreductase subunit beta	2.83 (0.66,3)	C-Fixation - in putative rTCA operon	WP_005965509.1	L0Y14_RS11980
KorC - 2-oxoacid:acceptor oxidoreductase	3.48 (0.71,3)	C-Fixation - in putative rTCA operon	WP_005965508.1	L0Y14_RS11985
HdrA- Heterodisulfide reductase, subunit A	3.45 (0.64,3)	Hdr-Flx- in putative rTCA operon	WP_005965507.1	L0Y14_RS11990
FlxD - Methyl-viologen-reducing hydrogenase	3.36 (0.67,3)	Hdr-Flx- in putative rTCA operon	WP_005965506.1	L0Y14_RS11995
FlxC- Coenzyme F420 hydrogenase/dehydrogenase	2.82 (1.32,4)	Hdr-Flx- in putative rTCA operon	WP_005965505.1	L0Y14_RS12000
FlxB- 4Fe-4S dicluster domain-containing protein	3.37 (0.80,3)	Hdr-Flx- in putative rTCA operon	WP_005965504.1	L0Y14_RS12005
FlxA- FAD/NAD(P)-binding protein	3.17 (0.63,3)	Hdr-Flx- in putative rTCA operon	WP_251859198.1	L0Y14_RS12010
AclB- ATP citrate lyase citrate, subunit B	3.47 (0.83,3)	C-Fixation - in putative rTCA operon	WP_040819861.1	L0Y14_RS12015
AclA- CoA-binding protein (ATP-citrate lyase, subunit A)	3.57 (0.80,3)	C-Fixation - in putative rTCA operon	WP_005962054.1	L0Y14_RS12020
Idh- NADP-dependent isocitrate dehydrogenase	3.42 (1.04,3)	C-Fixation - in putative rTCA operon	WP_006475384.1	L0Y14_RS12025
AcnA- aconitate hydratase	3.68 (0.71,3)	C-Fixation - in putative rTCA operon	WP_006475383.1	L0Y14_RS12030
PntA - Re/Si-specific NAD(P)(+) transhydrogenase subunit alpha	3.65 (0.64,3)	PntAB- in putative rTCA operon	WP_040819878.1	L0Y14_RS12035
PntB - Re/Si-specific NAD(P)(+) transhydrogenase subunit beta	3.53 (0.65,3)	PntAB- in putative rTCA operon	WP_040819860.1	L0Y14_RS12040
Mdh(1) - malate dehydrogenase	3.79 (0.47,3)	C-Fixation - in putative rTCA operon	WP_005962063.1	L0Y14_RS12045

Supplementary Table 5: Genes significant for sulfide condition in teal module.

Table of genes in teal module that were the most significant to the sulfide response. Selection criteria include genes with |GS| > 0.2 and p-value < 0.05, and |MM| > 0.8, both determined using Pearson correlations with two-tailed Student's p-values. These were further filtered for genes with significant differential expression (DE) with adjusted p-value (padj) < 0.05, determined via a robust two-sided linear model (limma package) with Benjamini-Hochberg adjustment for multiple comparisons. The average of these significant DE comparisons is reported below as mean logFC, with standard deviation and number of comparisons in parentheses. The sulfide comparisons where these genes were DE and thus used in these averages were: w_{vs}S(nO), s_{vs}S(nO), s_{vs}S(NO), H_{vs}S(NO), and H_{vs}S(nO).

Gene/product	mean logFC	Functional category	Accession	Locus ID
TorF family putative porin	-6.35 (0.88,4)	Import/export/transport	WP_006475174.1	L0Y14_RS00400
NorB(1) - Nitric oxide reductase large subunit	1.86 (0.53,3)	denitrification	WP_006475871.1	L0Y14_RS00515
NirS - nitrite reductase	2.52 (0.57,4)	denitrification	WP_005964485.1	L0Y14_RS00525
NorB(2) - nitric oxide reductase large subunit	1.86 (0.53,3)	denitrification	WP_006475871.1	L0Y14_RS00625
NorD(2) - Nitric oxide reductase activation protein	2.62 (0.77,4)	denitrification	WP_006475578.1	L0Y14_RS00655
PsrA – ribose-phosphate pyrophosphokinase	-2.42 (0.03,3)	nucleotide biosynthesis	WP_006475780.1	L0Y14_RS01660
fusA - elongation factor G	-1.99 (0.74,4)	translation	WP_040820199.1	L0Y14_RS01755
trmD - tRNA (guanine(37)-N(1))-methyltransferase	-1.94 (0.43,3)	translation	WP_005965409.1	L0Y14_RS02585
YqgE/AlgH family protein	2.38 (1.09,3)	possible regulatory	WP_005963206.1	L0Y14_RS03055
IbpA - Hsp20/alpha crystallin family protein	4.09 (1.56,4)	post translational modification	WP_005960924.1	L0Y14_RS04175
CTP synthase	-1.83 (0.53,4)	pyrimidine nucleotide biosynthesis	WP_006475214.1	L0Y14_RS04610
ftsH - ATP-dependent zinc metalloprotease	1.77 (0.73,4)	cell division, protein degradation	WP_240991664.1	L0Y14_RS04970
rimP - ribosome maturation factor	-2.39 (0.90,4)	ribosomal	WP_005965028.1	L0Y14_RS05075
nusA - transcription elongation factor	-2.38 (0.89,5)	transcription	WP_005965033.1	L0Y14_RS05080
fusA(2) - elongation factor G	2.18 (0.64,4)	translation	WP_006474122.1	L0Y14_RS06820
napG - ferredoxin-type protein NapG	2.65 (0.52,3)	periplasmic nitrate reductase	WP_005959809.1	L0Y14_RS06900
moaA - GTP 3',8-cyclase MoaA	1.90 (0.91,2)	cofactor biosynthesis	WP_005959805.1	L0Y14_RS06920
DUF4340 domain-containing protein	2.03 (0.28,2)	unknown	WP_006474714.1	L0Y14_RS07000
fusA(3) - elongation factor G	-1.67 (0.62,4)	translation	WP_005965298.1	L0Y14_RS07060
HolA-DNA polymerase III subunit delta	1.65 (0.22,3)	DNA synthesis	WP_050799404.1	L0Y14_RS07355
HupE - hydrogenase expression/formation protein	4.18 (NA,1)	[NiFe] Group 1e-hydrogenase assoc.	WP_005960737.1	L0Y14_RS07420
HyiA - hydrogenase small subunit	2.94 (2.05,4)	[NiFe] Group 1e-hydrogenase	WP_006475471.1	L0Y14_RS07425
Isp1 - cytochrome-like protein	4.19 (1.89,4)	[NiFe] Group 1e-hydrogenase	WP_005960739.1	L0Y14_RS07430
Isp2 - (Fe-S)-binding protein	3.88 (1.44,4)	[NiFe] Group 1e-hydrogenase	WP_006475472.1	L0Y14_RS07435
HyiB - nickel-dependent hydrogenase large subunit	3.57 (1.59,4)	[NiFe] Group 1e-hydrogenase	WP_040819914.1	L0Y14_RS07440
hypothetical	2.99 (NA,1)	likely [NiFe] Group 1e-hydrogenase assoc.	WP_010086938.1	L0Y14_RS07445
FrdB - SQOR family type B, subunit B	5.48 (1.11,4)	carbon metabolism	WP_005965241.1	L0Y14_RS07695
FrdC - SQOR family type B, subunit C	3.64 (0.86,4)	carbon metabolism	WP_005965237.1	L0Y14_RS07705
htpX - zinc metalloprotease	3.31 (0.73,4)	heat shock	WP_005958739.1	L0Y14_RS09150
cmoB - tRNA (mo5U34)-methyltransferase	1.77 (0.46,3)	post translational modification	WP_006473707.1	L0Y14_RS09305
hypothetical	-1.03 (NA,1)	unknown	WP_251859187.1	L0Y14_RS10325
ferritin family protein	3.80 (0.05,3)	unknown	WP_006475063.1	L0Y14_RS10635
KorB(2) - 2-oxoacid:ferredoxin oxidoreductase subunit beta	2.23 (0.94,3)	OGOR- carbon metabolism	WP_006475064.1	L0Y14_RS10640
KorA(2) - 2-oxoacid:acceptor oxidoreductase subunit alpha	2.43 (0.76,3)	OGOR- carbon metabolism	WP_039960060.1	L0Y14_RS10645
rhlE - ATP-dependent RNA helicase	-2.10 (0.76,4)	ATP-dependent RNA helicase RhlE	WP_006473886.1	L0Y14_RS10885
DUF2058 domain-containing protein	1.54 (0.46,3)	unknown	WP_005958947.1	L0Y14_RS10890
dnaJ - molecular chaperone	2.20 (0.77,4)	translation	WP_006473933.1	L0Y14_RS11230
PFOR(3) - pyruvate:ferredoxin (flavodoxin) oxidoreductase	2.09 (0.95,3)	PFOR-carbon metabolism	WP_005963105.1	L0Y14_RS13395
hypothetical	3.84 (1.37,4)	unknown	WP_005959787.1	L0Y14_RS13700
IspB - octaprenyl diphosphate synthase	-2.20 (1.28,4)	Ubiquinone/menaquinone synthesis	WP_005960787.1	L0Y14_RS14135
OMBP - Outer membrane beta-barrel protein	2.65 (1.13,5)	Import/export/transport	WP_005960810.1	L0Y14_RS14250
clpB - ATP-dependent chaperone	3.22 (1.23,5)	molecular chaperone	WP_006474487.1	L0Y14_RS14255
DUF4126 domain-containing protein	2.35 (0.82,3)	unknown	WP_005961677.1	L0Y14_RS14690
rho - transcription termination factor	-2.23 (0.04,3)	transcription	WP_006474897.1	L0Y14_RS15135
ptsP- phosphoenolpyruvate--protein phosphotransferase	1.33 (0.22,4)	sugar uptake	WP_006474659.1	L0Y14_RS15555
feoB - Fe(2+) transporter permease subunit FeoB	1.53 (0.38,3)	import/export/transport	WP_006474641.1	L0Y14_RS15640
McrA - penicillin-binding protein 1A	-1.37 (NA,1)	cell wall	WP_006475433.1	L0Y14_RS16020
KorA(3) - 2-oxoacid:acceptor oxidoreductase subunit alpha	-2.62 (0.80,5)	OGOR- carbon metabolism	WP_240991706.1	L0Y14_RS16060
KorB(3) - 2-oxoglutarate ferredoxin oxidoreductase subunit beta	-2.57 (0.41,4)	OGOR- carbon metabolism	WP_005958509.1	L0Y14_RS16065
PFOR-like ferredoxin	-1.91 (0.52,4)	possible PFOR-carbon metabolism	WP_006475426.1	L0Y14_RS16070

Supplementary Table 6: Genes significant for oxygen condition in purple module.

Table of genes in purple module that were the most significant to the oxygen response. Selection criteria include genes with |GS| > 0.2 and p-value < 0.05, and |MM| > 0.8, both determined using Pearson correlations with two-tailed Student's p-values. These were further filtered for genes with significant differential expression (DE) with adjusted p-value (padj) < 0.05, determined via a robust two-sided linear model (limma package) with Benjamini-Hochberg adjustment for multiple comparisons. The average of these significant DE comparisons is reported below as mean logFC, with standard deviation and number of comparisons in parentheses. The oxygen comparisons where these genes were DE and thus used in these averages were: o_{vs}O(SN) and o_{vs}O(Hn).

Gene/product	Mean logFC	Functional category	Accession	Locus ID
hypothetical	-3.85 (0.27,2)	possible transcriptional regulator	WP_040818945.1	L0Y14_RS05880
GlcD - FAD-binding oxidoreductase	-4.79 (0.72,2)	unknown	WP_005964373.1	L0Y14_RS06635
fusA(2) - elongation factor G	2.30 (0.17,2)	translation	WP_006474122.1	L0Y14_RS06820
hypE - hydrogenase expression/formation protein HypE	2.42 (0.11,2)	[NiFe] Group 1e-hydrogenase assoc.	WP_005965304.1	L0Y14_RS07045
hypothetical	4.01 (1.56,2)	likely [NiFe] Group 1e-hydrogenase assoc.	WP_006474708.1	L0Y14_RS07050
HupE - hydrogenase expression/formation protein	3.16 (0.17,2)	[NiFe] Group 1e-hydrogenase assoc.	WP_005960737.1	L0Y14_RS07420
HyiA - hydrogenase small subunit	4.75 (0.90,2)	[NiFe] Group 1e-hydrogenase	WP_006475471.1	L0Y14_RS07425
Isp2 - (Fe-S)-binding protein	4.24 (1.49,2)	[NiFe] Group 1e-hydrogenase	WP_006475472.1	L0Y14_RS07435
VWA domain-containing protein	3.87 (NA,1)	unknown	WP_251859293.1	L0Y14_RS07495
FBP- Flagellar brake protein	-4.94 (NA,1)	motility	WP_050799358.1	L0Y14_RS08165
ppdk - pyruvate, phosphate dikinase	1.96 (0.03,2)	carbon metabolism	WP_006473784.1	L0Y14_RS08840
CvpA family protein	-6.82 (1.32,2)	possible antibiotic production	WP_005960134.1	L0Y14_RS09775
SgpA(1)- sulfur globule protein	4.70 (0.28,2)	elemental sulfur storage	WP_081417773.1	L0Y14_RS11490
RocR- sigma 54-interacting transcriptional regulator	2.76 (1.02,2)	transcriptional regulator	WP_005966820.1	L0Y14_RS11685
hypothetical	-4.71 (1.66,2)	unknown	WP_005959002.1	L0Y14_RS12695
CphA- cyanophycin synthetase	-4.85 (0.06,2)	nitrogen and carbon storage	WP_251859203.1	L0Y14_RS12700
PFOR(3) - pyruvate:ferredoxin (flavodoxin) oxidoreductase	2.20 (0.60,2)	PFOR-carbon metabolism	WP_005963105.1	L0Y14_RS13395
hypothetical	2.46 (0.58,2)	unknown	WP_006474741.1	L0Y14_RS13990
glycoside hydrolase family 16 protein	-3.17 (0.51,2)	Polysaccharide Degradation	WP_006474472.1	L0Y14_RS14145
BamD- outer membrane protein assembly factor	-2.11 (0.08,2)	membrane	WP_005960797.1	L0Y14_RS14185
RimN- L-threonylcarbamoyladenylate synthase	-5.70 (0.76,2)	translation	WP_005966228.1	L0Y14_RS15690
McrA - penicillin-binding protein 1A	-1.40 (0.06,2)	cell wall	WP_006475433.1	L0Y14_RS16020
yidC - membrane protein insertase	-2.87 (0.70,2)	membrane	WP_039959545.1	L0Y14_RS16345
yidD - membrane protein insertion efficiency factor	-7.25 (NA,1)	membrane	WP_006474993.1	L0Y14_RS16350

Supplementary Table 7: Genes significant for oxygen condition in black module.

Table of genes in black module that were the most significant to the oxygen response. Selection criteria include genes with |GS| > 0.2 and p-value < 0.05, and |MM| > 0.8, both determined using Pearson correlations with two-tailed Student's p-values. These were further filtered for genes with significant differential expression (DE) with adjusted p-value (padj) < 0.05, determined via a robust two-sided linear model (limma package) with Benjamini-Hochberg adjustment for multiple comparisons. Out of 33 genes that met the first parameter, 27 were also significantly DE in one of these comparisons. The average of these significant DE comparisons is reported below as mean logFC, with standard deviation and number of comparisons in parentheses. The oxygen comparisons where these genes were DE and thus used in these averages were: o_{vs}O(SN) and o_{vs}O(Hn).

Gene/product	Mean logFC	Functional category	Accession	Locus ID
Hr - bacteriohemerythrin	-2.53 (0.67,2)	oxygen binding	WP_005966420.1	L0Y14_RS00450
hypothetical	-2.63 (NA,1)	unknown	WP_006474341.1	L0Y14_RS01145
ftsE - cell division ATP-binding protein	-3.07 (NA,1)	cell division	WP_039959456.1	L0Y14_RS02180
hypothetical	-4.50 (NA,1)	unknown	WP_039960219.1	L0Y14_RS03075
fabA - 3-hydroxyacyl-[acyl-carrier-protein] dehydratase	-3.61 (NA,1)	fatty acid synthesis	WP_005961657.1	L0Y14_RS03375
HIT-like - DUF4931 domain-containing protein	-4.80 (NA,1)	nucleotide processing	WP_006475324.1	L0Y14_RS03820
CbbQ - CbbQ/NirQ/NorQ/GpvN family protein	-3.51 (NA,1)	possible RuBisCO regulator	WP_005960013.1	L0Y14_RS04045
hypothetical	-3.85 (0.27,2)	possible transcriptional regulator	WP_040818945.1	L0Y14_RS05880
transglycosylase - SLT domain-containing protein	-2.94 (NA,1)	cell wall	WP_138921748.1	L0Y14_RS06585
acnB - aconitate hydratase	-1.48 (NA,1)	TCA-carbon metabolism	WP_006474097.1	L0Y14_RS06620
IhfB - integration host factor subunit beta	-4.48 (NA,1)	DNA regulation	WP_005963820.1	L0Y14_RS06760
hypothetical	-4.99 (NA,1)	unknown	WP_005964816.1	L0Y14_RS08400
class I SAM-dependent methyltransferase	-3.51 (NA,1)	unknown	WP_006473786.1	L0Y14_RS08830
prsK - PEP-CTERM system histidine kinase	-2.65 (NA,1)	signal transduction	WP_240991685.1	L0Y14_RS09840
FliK - flagellar hook-length control protein	-3.23 (NA,1)	motility	WP_006473885.1	L0Y14_RS10875
malQ - 4-alpha-glucanotransferase	1.41 (NA,1)	glycogen synthesis/degradation	WP_005965378.1	L0Y14_RS11140
hypothetical	-3.41 (NA,1)	unknown	WP_005962035.1	L0Y14_RS11330
SIS-GmHa - SIS domain-containing protein	-3.34 (NA,1)	membrane biosynthesis	WP_005959007.1	L0Y14_RS12670
TIGR04211 family SH3 domain-containing protein	-3.91 (1.22,2)	unknown	WP_006475744.1	L0Y14_RS13180
rpmB - 50S ribosomal protein L28	-4.41 (2.08,2)	ribosomal	WP_005959883.1	L0Y14_RS14085
hypothetical	-6.17 (NA,1)	unknown	WP_251859220.1	L0Y14_RS14595
PG peptidase- peptidoglycan DD-metalloendopeptidase	-3.82 (NA,1)	cell wall	WP_006475071.1	L0Y14_RS14660
IscA - iron-sulfur cluster assembly accessory protein	-2.76 (NA,1)	post translational modification	WP_006475076.1	L0Y14_RS14700
LolA-like - outer membrane lipoprotein-sorting protein	-3.51(1.48,2)	membrane	WP_005959981.1	L0Y14_RS14970
chalcone isomerase family protein	-2.86 (NA,1)	unknown	WP_240991636.1	L0Y14_RS15890
PRX-BCP - peroxiredoxin	-3.32 (NA,1)	oxidative stress	WP_006475445.1	L0Y14_RS15950
Dut - dUTP diphosphatase	-3.00 (NA,1)	DNA maintenance	WP_006475014.1	L0Y14_RS16130
