# Supplementary material for: Co-expression analysis reveals distinct alliances around two carbon fixation pathways in hydrothermal vent symbionts
Source: Nat Microbiol. 2024 Jun 5;9(6):1526–39. doi: 10.1038/s41564-024-01704-y (PMC11636981; doi:10.1038/s41564-024-01704-y)
Supplement: Supplementary file 2 — Reporting Summary [file 41564_2024_1704_MOESM2_ESM.pdf]

nature portfolio

Corresponding author(s): Girguis, Peter and Mitchell, Jessica
Last updated by author(s): March 27, 2024

Reporting Summary

Nature Portfolio wishes to improve the reproducibility of the work that we publish. This form provides structure for consistency and transparency in reporting. For further information on Nature Portfolio policies, see our Editorial Policies and the Editorial Policy Checklist.

Statistics

For all statistical analyses, confirm that the following items are present in the figure legend, table legend, main text, or Methods section.

n/a	Confirmed	
☐	☒	The exact sample size (*n*) for each experimental group/condition, given as a discrete number and unit of measurement
☐	☒	A statement on whether measurements were taken from distinct samples or whether the same sample was measured repeatedly
☐	☒	The statistical test(s) used AND whether they are one- or two-sided<br>*Only common tests should be described solely by name; describe more complex techniques in the Methods section.*
☐	☒	A description of all covariates tested
☐	☒	A description of any assumptions or corrections, such as tests of normality and adjustment for multiple comparisons
☐	☒	A full description of the statistical parameters including central tendency (e.g. means) or other basic estimates (e.g. regression coefficient) AND variation (e.g. standard deviation) or associated estimates of uncertainty (e.g. confidence intervals)
☐	☒	For null hypothesis testing, the test statistic (e.g. *F*, *t*, *r*) with confidence intervals, effect sizes, degrees of freedom and *P* value noted<br>*Give P values as exact values whenever suitable.*
☒	☐	For Bayesian analysis, information on the choice of priors and Markov chain Monte Carlo settings
☐	☒	For hierarchical and complex designs, identification of the appropriate level for tests and full reporting of outcomes
☐	☒	Estimates of effect sizes (e.g. Cohen's *d*, Pearson's *r*), indicating how they were calculated

Our web collection on statistics for biologists contains articles on many of the points above.

Software and code

Policy information about availability of computer code

Data collection No software was used to data collection.

Data analysis	FastQC v.0118 (www.bioinformatics.babraham.ac.uk/projects/fastqc/)<br>TrimGalore! v0.6.5 (www.bioinformatics.babraham.ac.uk/projects/trim_galore/)<br>R v4.0.0-v4.3.3<br>Bowtie2 2.5.3<br>ggplot2 v3.2.0 -3.5.0<br>https://github.com/harvardinformatics/TranscriptomeAssemblyTools<br>RSEM v.1.3.1<br>limma v3.17<br>WGCNA v1.72<br>Cytoscape v. 3.9.1<br>cyto-Hubba v. 0.1<br>UpsetR v1.4.0<br>HydDB (https://services.birc.au.dk/hyddb/)<br>DeepTMHMM (https://dtu.biolib.com/DeepTMHMM)<br>HMMR (https://www.ebi.ac.uk/Tools/hmmer/)<br>STRING (https://string-db.org/)<br>MetalPredator (http://metalweb.cerm.unifi.it/tools/metalpredator/)<br>NCBI BLAST<br>NCBI COBALT<br>CD search (www.ncbi.nlm.nih.gov/home/analyze/)

For manuscripts utilizing custom algorithms or software that are central to the research but not yet described in published literature, software must be made available to editors and reviewers. We strongly encourage code deposition in a community repository (e.g. GitHub). See the Nature Portfolio guidelines for submitting code & software for further information.

Data

Policy information about availability of data

All manuscripts must include a data availability statement. This statement should provide the following information, where applicable:

- Accession codes, unique identifiers, or web links for publicly available datasets
- A description of any restrictions on data availability
- For clinical datasets or third party data, please ensure that the statement adheres to our policy

Raw sequencing data have been submitted to the NCBI Sequence Read Archive (SRA). SRA: SRP323622. Project ID: PRJNA736714; https://www.ncbi.nlm.nih.gov/bioproject/PRJNA736714. Processed data files (read counts, differential expression, and co-expression analyses) have been deposited in NCBI's Gene Expression Omnibus and are accessible through GEO Series accession number GSE249345; https://www.ncbi.nlm.nih.gov/geo/query/acc.cgi?acc=GSE249345. All other data are available in the supplementary material, and source data files.

Research involving human participants, their data, or biological material

Policy information about studies with human participants or human data. See also policy information about sex, gender (identity/presentation), and sexual orientation and race, ethnicity and racism.

Reporting on sex and gender	N/A
Reporting on race, ethnicity, or other socially relevant groupings	N/A
Population characteristics	N/A
Recruitment	N/A
Ethics oversight	N/A

Note that full information on the approval of the study protocol must also be provided in the manuscript.

Field-specific reporting

Please select the one below that is the best fit for your research. If you are not sure, read the appropriate sections before making your selection.

☐ Life sciences ☐ Behavioural & social sciences ☒ Ecological, evolutionary & environmental sciences

For a reference copy of the document with all sections, see nature.com/documents/nr-reporting-summary-flat.pdf

Ecological, evolutionary & environmental sciences study design

All studies must disclose on these points even when the disclosure is negative.

Study description	Deep sea hydrothermal vent tubeworms were collected and kept alive at experimental conditions for studying the effect of the external environmental factors (e.g. dissolved gases) on the metabolism of their chemoautotrophic symbionts.

Research sample	30 deep sea hydrothermal tubeworms (Riftia pachyptila) were collected and incubated at in situ pressures and temperatures. During the course of the incubations, aquaria water samples were analyzed for changes in dissolved oxygen, hydrogen, sulfide, dissolved inorganic carbon and pH. The tubeworms were dissected and samples of their trophosome (the symbiont bearing tissue found deep within the worm) were taken for RNA and isotope analyses. Other tissue samples of the worms were also taken for isotope analyses.
Sampling strategy	Riftia pachyptila were collected using a human operated vehicle (HOV Alvin) aboard R/V Atlantis. We chose worms ~1ft in length or less so that they would fit in our pressurized aquaria. Worms were collected at the end of the dive with the HOV and upon surfacing, were brought back to pressure within 1-2 hours. Each aquaria held 4-5 worms, in flow through conditions, with active stirring.
Data collection	Riftia pachyptila were collected with the submersible Alvin (operated by the pilot) and sampling directions were given by Jessica Mitchell, and site information was recorded for each collection.
Timing and spatial scale	Riftia pachyptila were collected during two research cruises to the East Pacific Rise on 02-21 Novemeber 2014 and 08-28 October 2016. Riftia were collected from the vent sites, "Crab Spa", "Tica" and "Bio9" during multiple collections.
Data exclusions	Worms that did not present as healthy after incubations were removed from these analyses. Our criteria for healthy are that they must be responsive to touch, responsive to more acute stimuli (e.g., being poked with a toothpick), must not show any body lesions, and must not have an off-odor consistent with tissue necrosis.
Reproducibility	All computational analyses were conducted using open source softwares with versions and any flags specified and can be reproduced accordingly.
Randomization	A stratified randomization technique was employed when selecting Riftia for incubations such that, each vessel got a similar size distribution of worms.
Blinding	Blinding is not relevant for this study because the aim of the study is to characterize and compare the shifts in microbial metabolism under controlled environmental conditions.

Did the study involve field work? ☒ Yes ☐ No

Field work, collection and transport

Field conditions	Field work was conducted aboard R/V Atlantis in the East Pacific Rise. Weather reports for each day are available at the RCR repository. Briefly, all samples were collected on clear days with no documented precipitation.
Location	Riftia pachyptila were collected during two research expeditions on the R/V Atlantis to the East Pacific Rise using the HOV Alvin in 2014 and 2016 at the following coordinates: 27.40921631°N, 111.38910334°W. water depth was 1810 meters.
Access & import/export	All samples were collected under the appropriate permits, which principally includes US Fish and Wildlife and USDA permits. No animals fall under CITES or the Nagoya protocol
Disturbance	Our collection methods are consistent with best practices in the field, and result in minimal or no damage to the vent site when sampling and only animals that were needed for the study were collected.

Reporting for specific materials, systems and methods

We require information from authors about some types of materials, experimental systems and methods used in many studies. Here, indicate whether each material, system or method listed is relevant to your study. If you are not sure if a list item applies to your research, read the appropriate section before selecting a response.

Materials & experimental systems

n/a	Involved in the study	
☒	☐	Antibodies
☒	☐	Eukaryotic cell lines
☒	☐	Palaeontology and archaeology
☒	☐	Animals and other organisms
☒	☐	Clinical data
☒	☐	Dual use research of concern
☒	☐	Plants

Methods

n/a	Involved in the study	
☒	☐	ChIP-seq
☒	☐	Flow cytometry
☒	☐	MRI-based neuroimaging
